# Effectiveness of pharmacological treatments for severe agitation in real-world emergency settings: protocol of individual-participant-data network meta-analysis

**DOI:** 10.1186/s13643-024-02623-z

**Published:** 2024-08-02

**Authors:** Spyridon Siafis, Hui Wu, Nobuyuki Nomura, Johannes Schneider-Thoma, Irene Bighelli, Carolin Lorenz, Joseph E. Dib, Prathap Tharyan, Leonie A. Calver, Geoffrey K. Isbister, Esther W. Y. Chan, Jonathan C. Knott, Celene Y. L. Yap, Célia Mantovani, Marc L. Martel, David Barbic, William G. Honer, Wulf-Peter Hansen, Gisele Huf, Jacob Alexander, Nirmal S. Raveendran, Evandro S. F. Coutinho, Josef Priller, Clive E. Adams, Georgia Salanti, Stefan Leucht

**Affiliations:** 1 Technical University of Munich, TUM School of Medicine and Health, Department of Psychiatry and Psychotherapy, Klinikum rechts der Isar, Munich, Germany; 2 German Center for Mental Health (DZPG), Partner site München/Augsburg, Munich, Germany; 3 Department of Neuropsychiatry, Keio University School of Medicine, Tokyo, Japan; 4 Division of Psychiatry & Applied Psychology, Institute of Mental Health, School of Medicine, University of Nottingham, Nottingham, Nottinghamshire UK; 5 Clinical Epidemiology Unit, Christian Medical Centre, Vellore, India; 6 School of Nursing and Midwifery, University of Newcastle, Callaghan, NSW Australia; 7 Clinical Toxicology Research Group, University of Newcastle, Newcastle, NSW Australia; 8 Department of Pharmacology and Pharmacy, University of Hong Kong, Pok Fu Lam, Hong Kong SAR, China; 9 Laboratory of Data Discovery for Health (D24H), Science Park, Hong Kong SAR, China; 10 Department of Pharmacy, HKU-Shenzhen Hospital, Shenzhen, China; 11 HKU-SZ Institute of Research and Innovation (SIRI), Shenzhen, China; 12 Department of Critical Care, The University of Melbourne, Melbourne, VIC Australia; 13 Department of Nursing, Faculty of Medicine, Dentistry and Health Sciences, Melbourne School of Health Sciences, The University of Melbourne, Melbourne, VIC Australia; 14 Department of Neurosciences and Behavior, Ribeirao Preto School of Medicine, Universidade de Sao Paulo, São Paulo, Brazil; 15 Department of Emergency Medicine, Hennepin County Medical Center, Minneapolis, MN USA; 16 Department of Emergency Medicine, University of British Columbia, Vancouver, BC Canada; 17 Centre for Health Evaluation & Outcomes Sciences, St. Paul’s Hospital, Vancouver, BC Canada; 18 Department of Psychiatry, Faculty of Medicine, University of British Columbia, Vancouver, BC Canada; 19 BC Mental Health and Substance Use Services Research Institute, Vancouver, BC Canada; 20 BASTA-Das Bündnis für psychisch erkrankte Menschen, Munich, Germany; 21 National Institute of Quality Control in Health, Oswaldo Cruz Foundation, Av. Brasil 4365, Manguinhos, Rio de Janeiro, Brazil; 22 SA Health, Adelaide, Australia; 23 Department of Psychiatry, Christian Medical College, Vellore, Tamil Nadu India; 24 Department of Epidemiology, Institute of Social Medicine, State University of Rio de Janeiro, Rio de Janeiro, Brazil; 25 Neuropsychiatry and Laboratory of Molecular Psychiatry, Charité - Universitätsmedizin Berlin and DZNE, Berlin, Germany; 26 University of Edinburgh and UK DRI, Edinburgh, UK; 27 Institute of Mental Health, University of Nottingham, Nottingham, Nottinghamshire UK; 28 Institute of Social and Preventive Medicine (ISPM), University of Bern, Bern, Switzerland

**Keywords:** Aggression, Agitation, Violence, Effectiveness, Psychosis, Tranquilization, Emergency, Systematic review, Individual-participant data, Network meta-analysis

## Abstract

**Background:**

Severe psychomotor agitation and aggression often require immediate pharmacological intervention, but clear evidence-based recommendations for choosing among the multiple options are lacking. To address this gap, we plan a systematic review and individual-participant-data network meta-analysis to investigate their comparative effectiveness in real-world emergency settings with increased precision.

**Methods:**

We will include randomized controlled trials investigating intramuscular or intravenous pharmacological interventions, as monotherapy or in combination, in adults with severe psychomotor agitation irrespective of the underlying diagnosis and requiring rapid tranquilization in general or psychiatric emergency settings. We will exclude studies before 2002, those focusing on specific reasons for agitation and placebo-controlled trials to avoid concerns related to the transitivity assumption and potential selection biases. We will search for eligible studies in BIOSIS, CENTRAL, CINAHL Plus, Embase, LILACS, MEDLINE via Ovid, PubMed, ProQuest, PsycINFO, ClinicalTrials.gov, and WHO-ICTRP. Individual-participant data will be requested from the study authors and harmonized into a uniform format, and aggregated data will also be extracted from the studies. At least two independent reviewers will conduct the study selection, data extraction, risk-of-bias assessment using RoB 2, and applicability evaluation using the RITES tool. The primary outcome will be the number of patients achieving adequate sedation within 30 min after treatment, with secondary outcomes including the need for additional interventions and adverse events, using odds ratios as the effect size. If enough individual-participant data will be collected, we will synthesize them in a network meta-regression model within a Bayesian framework, incorporating study- and participant-level characteristics to explore potential sources of heterogeneity. In cases where individual-participant data are unavailable, potential data availability bias will be explored, and models allowing for the inclusion of studies reporting only aggregated data will be considered. We will assess the confidence in the evidence using the Confidence in Network Meta-Analysis (CINeMA) approach.

**Discussion:**

This individual-participant-data network meta-analysis aims to provide a fine-tuned synthesis of the evidence on the comparative effectiveness of pharmacological interventions for severe psychomotor agitation in real-world emergency settings. The findings from this study can greatly be provided clearer evidence-based guidance on the most effective treatments.

**Systematic review registration:**

PROSPERO CRD42023402365.

**Supplementary Information:**

The online version contains supplementary material available at 10.1186/s13643-024-02623-z.

## Background

Psychomotor agitation is a common medical emergency characterized by inner tension and excessive motor activity [1, 2]. While milder forms can be managed with less invasive interventions like de-escalation techniques, severe agitation poses a heightened risk of harm to the patient, hospital staff, and others. In such scenarios, rapid pharmacological intervention with intramuscular or intravenous drugs becomes essential to quickly calm the patient and, ideally, address the underlying condition without causing oversedation or other adverse events [2, 3].

Multiple pharmacological options are available, including various first- and second-generation antipsychotics, benzodiazepines, either alone or in combination. These options can differ importantly in their effectiveness and risk-to-benefit ratios. Despite this, there are no clear, evidence-based recommendations on the most appropriate treatments, leading to inconsistencies across guidelines [2–9]. For example, the Australian schizophrenia guideline recommends intramuscular olanzapine as the first choice and droperidol as the second [9], while the German S3 schizophrenia guideline recommends parenteral lorazepam as first and antipsychotics as second [4]. These discrepancies underscore the lack of consensus and the need for more robust evidence to guide clinical practice and optimize management strategies.

The complexity of this issue is further compounded by the fact that, until recently, few trials have been conducted in real-world emergency settings with severely agitated participants of various underlying etiologies, who often cannot provide informed consent. Such trials are crucial to generating applicable evidence in this area. Notable global efforts include the paradigmatic TREC trials (Tranquilização Rápida-Ensaio Clínico; Rapid Tranquillization-Clinical Trial) [10–14], and several subsequent trials [15–17], which feature pragmatic designs and large sample sizes. Despite the existence of these individual trials, previous systematic reviews on this topic did not focus on emergency settings, making their generalizability uncertain [18–22]. One network meta-analysis, which can compare all available pharmacological interventions even if not directly compared in single trials, excluded psychiatric emergency departments, where agitation is frequent [23]. Furthermore, these meta-analyses were based on study-level data, lacking the increased power and detailed information of individual-participant data needed to generate more precise estimates and examine potential subgroup differences in this heterogeneous condition [24, 25].

Currently, there is no individual-participant-data network meta-analysis that synthesized the evidence from the relevant clinical trials mentioned above. This highlights the need for a comprehensive and fine-grained evidence synthesis to provide more definitive answers and inform treatment decisions for severe agitation in real-world emergency settings.

### Objectives

To address this critical gap, we plan a systematic review and individual participant data network meta-analysis of clinical trials to examine the effectiveness and tolerability of intramuscular or intravenous pharmacological interventions for severe psychomotor agitation in general and psychiatric emergency departments.

By synthesizing data from trials conducted in real-world settings and integrating individual participant data with network meta-analysis, we aim to overcome the limitations of previous reviews and provide more precise and applicable information. This approach will enable us to address the heterogeneity of the condition and explore potential subgroup differences, ultimately contributing to the development of more uniform and evidence-based guidelines.

## Methods

The protocol of this systematic review was registered to PROSPERO on March 9, 2023 (ID: CRD42023402365) and reported according to the PRISMA statement extension for protocols (PRISMA-P) (eAppendix-1) [26]. The status of the review at the time of submission and any modifications made from the initial version of the protocol are outlined in eAppendix-2. If further amendments to the protocol are necessary, we will update the PROSPERO registration and provide clear reporting of any deviations from the original protocol in the published manuscript.

### Eligibility criteria

#### Participants

We will include adults (as defined in the original studies) with acute and severe psychomotor agitation and/or aggression requiring parenteral pharmacological intervention in emergency settings (see the “Study design and setting”), irrespective of the underlying diagnosis, diagnostic criteria, sex, and ethnicity (see also eAppendix-3 for a table of the inclusion and exclusion criteria).

Eligible patients are expected to be unable to provide informed consent [27], and thus, we will exclude studies conducted in settings requiring informed consent by the patient themselves before treatment (see also “Interventions” and “Study design and setting”), such as industry-sponsored studies designed for regulatory purposes [28]. Including these studies would lead to important selection bias and undermine the generalizability of the findings.

Moreover, severe agitation is a transdiagnostic condition, and pharmacological intervention often precedes formal diagnostic procedures [2]. For this reason, we will not restrict to any diagnostic group, and trials with broad inclusion criteria regarding the causes of agitation or aggression will be eligible. Nevertheless, we will exclude trials focusing on specific reasons of agitation by their inclusion criteria such as studies focusing on delirium or dementia (for which antipsychotics are generally contraindicated). The inclusion of such studies would pose serious concerns to the transitivity assumption for the network meta-analysis (see the “Data synthesis”) [29]. Similarly, we will exclude studies focusing on children, adolescents [30], and patients of advanced age as defined by the inclusion criteria of the original studies. However, we will not further exclude participants based on their age at the IPD level.

#### Interventions

We will include drugs administered intramuscularly or intravenously, either as monotherapy or in combination, to calm patients with severe agitation, i.e., antipsychotics (e.g., haloperidol, droperidol, olanzapine, aripiprazole, ziprasidone, clotiapine, chlorpromazine, promethazine), benzodiazepines (e.g., lorazepam, midazolam, diazepam), antihistamines (e.g., diphenhydramine), alpha_2_ adrenergic agonists (e.g., clonidine, dexmedetomidine), and ketamine. The intramuscular and intravenous formulations of the same drug will be regarded as distinct interventions. A combination of eligible drugs will be considered as a new eligible intervention. There will also be no restriction in terms of dose, which will be included as a covariate in the analysis (see “Data synthesis”). We will exclude barbiturates as they are no longer used for this indication due to their narrow therapeutic window. We will also exclude studies with oral and inhaled formulations given that these routes of administration require cooperative patients [2], who would not have been eligible for our analysis (see “Participants”). Moreover, we will exclude studies that used placebo, due to the ethical concerns surrounding its use in emergency settings and the potential for selection bias in such trials.

#### Comparison groups

There is no single comparison group in a network meta-analysis, given that all eligible experimental interventions will be compared with each other. Yet, we will use intramuscular haloperidol as a reference in the forest plots (see “Data synthesis”) because it is a widely accessible and frequently used drug for agitation [19].

#### Outcomes

The outcomes were selected in accordance with the design of the TREC trials [10–14], which were based on early consultation with frontline clinicians in busy state hospital psychiatric emergency settings. This selection aimed to cover a broad range of effectiveness, acceptability, tolerability, and service use outcomes (see also a list of outcomes in eAppendix-4).

### Primary outcome

Our primary outcome will be the proportion of patients with adequate sedation achieved in each study arm within 30 min after the first administration of the intervention (preferably as close as possible to 20 min). This time-point was selected as primary because severe agitation is a clinical emergency that should be treated as quickly as possible in real-world settings, and it was identified as highly relevant in previous studies [10–14, 23]. Moreover, we will also analyze time to adequate sedation (see below), and secondary time-points will include 10 min, 30 min, 45 min, 60 min, 2 h, 4 h, and 24 h after the first administration of the intervention.

The trials may use various methods to define adequate sedation, including clinical judgement or cutoffs of various rating scales, e.g., Richards’ Sedation Scale [31] or Sedation Assessment Tool (SAT) [32]. If the available data allow, we will aim to apply relatively homogeneous cutoffs and definitions of adequate sedation, i.e., “calm” or “asleep” but ideally not oversedated. Any decisions on choosing the most appropriate definition in each study will be documented and made in consultation with experts in this field (e.g., authors of the original studies who have agreed to join the review team). Nonetheless, according to a previous systematic review, definitions of adequate sedation are expected to be similar across studies and rating scales [23]. We will also use relative effect size indices, which are not anticipated to be substantially affected by potentially different definitions (see “Data synthesis”) [33].

### Secondary outcomes

We will also collect data on the following secondary outcomes, if available:

1. Time to adequate sedation, as defined above
2. The proportion of patients requiring additional pharmacological intervention, e.g., an additional dose of the same or another medication
3. The proportion of patients requiring physical restraints
4. Mean scores of rating scales measuring the severity of agitated behavior, e.g., Positive and Negative Syndrome Scale Excitement Component (PANSS-EC) [34] and Overt Aggression Scale (OAS) [35]. However, implementing these rating scales can be difficult in real-world emergency settings, and thus, they may only be available in some of the eligible trials.
5. The proportion of patients discharged from the hospital or the emergency setting. Although hospital discharge is an important service use outcome measured in previous studies [10–14], it may heavily depend on other factors such as accessibility rather than the initial intervention taken.
6. The proportion of patients with important adverse events, i.e., seizures, dystonia, akathisia, parkinsonism, any extrapyramidal side effect, use of antiparkinsonian medications, QTc interval prolongation and arrhythmias, falls, respiratory depression, aspiration, allergic reaction, bronchospasm, oversedation, hypotension, nausea, and vomiting. The adverse events could be reported in various ways across trials, and we will aim to harmonize them using the Medical Dictionary for Regulatory Activities (MedDRA) terminology [36].
7. Mean scale scores of the severity of extrapyramidal side effects, e.g., Simpson-Angus Scale (SAS) [37] and Barnes Akathisia Rating Scale (BARS) [38]
8. The proportion of patients that died due to any reason
9. The proportion of patients with a serious adverse event [39]
10. The proportion of patients that dropped out of the study due to any reason, ineffectiveness, and adverse events

Secondary outcomes will be examined within 30 min, 1 h, 2 h, 4 h, and 24 h after the first administration of the intervention, depending on the availability of data across studies. If longer-term data are available in the respective studies, they will be considered.

### Study design and setting

We will include randomized controlled trials (RCTs) that compared at least two drugs, at least two doses of the same drug, two different formulations of drugs, two different combinations of drugs, or combination of drugs versus monotherapy for severe agitation and aggression. We will include studies focusing on investigating rapid tranquilization by assessing sedation within 30 min after the intervention (see “Outcomes”), and a similar approach was used in a previous meta-analysis [23]. Eligible RCTs would be conducted in general or psychiatric emergency rooms or psychiatric emergency wards, where participants with severe psychomotor agitation requiring rapid tranquilization may not be able to provide informed consent before (see “Participants” and “Interventions”). Therefore, placebo-controlled RCTs and other studies requiring informed consent from participants prior to intervention will be excluded. RCTs in which the participants or legal guardians could provide informed consent after the intervention will be included. We will also exclude studies conducted in other specialized settings, such as palliative care, intensive, and critical care units (see “Participants”).

We will include both open and blinded (single- and double-blind) RCTs, but studies with a high risk of bias in the randomization process will be excluded (see the “Risk-of-bias assessment”). In crossover trials, we will use only the first phase to avoid carry-over effects as agitation is often resolved after the first treatment [40]. It is not expected that cluster randomized trials would be found, but in that case, we will consider the implications of clustering in extracting study treatment effects [41].

We will include studies since 2002, i.e., when the first paradigmatic TREC trials were conducted [10, 11]. This decision is also based on the difficulties in retrieving IPD from older trials of more than 20 years ago (e.g., data no longer available) [42] and the potential differences in the design and quality compared with more recent trials [43]. We will exclude studies whose publications have been retracted [44–46]. There will be no restrictions on the study eligibility criteria in terms of the language of publication and country of origin [47].

### Information sources and search strategy

We will search for eligible trials in multiple electronic databases, i.e., BIOSIS, CENTRAL, CINAHL Plus, Embase, LILACS, MEDLINE via Ovid, PubMed, ProQuest, PsycINFO, and the clinical trial registries ClinicalTrials.gov and WHO-ICTRP. There will be no restrictions on the search strategies in terms of language, publication status, and document type [47]. The search strategies will be developed in collaboration with an information specialist (see the “Acknowledgements”) using keywords for agitation, tranquilization treatment, emergency settings, and clinical trials as presented in eAppendix-5. The final search strategies for all databases will also be reported according to the PRISMA extension for reporting literature searches (PRISMA-S) [48]. We will also inspect the reference list of included studies and previous reviews [18–23].

We will contact the authors of eligible studies and pharmaceutical companies associated with eligible industry-sponsored studies, if identified, to request anonymized IPD and any additional relevant studies. E-mails will be sent to the first and/or corresponding author of the studies, and in the event of nonresponse, we will send reminders and attempt to contact other authors or use alternative communication methods such as telephone. If there are persisting uncertainties regarding the eligibility criteria and/or IPD availability due to inadequate author responses despite our efforts, we will classify the study as “awaiting classification” and exclude the study from the analysis.

### Study selection and data collection

#### Study selection

Two independent reviewers will perform a two-step screening process to identify eligible studies from the records obtained in the search. In the first step, they will assess the titles/abstracts to identify potentially relevant studies. In the second step, full texts of potentially relevant or unclear records will be obtained, and the reviewers will assess them against the eligibility criteria. Any disagreements between the two reviewers will be resolved through consultation with a third senior reviewer. In cases where further information is needed, the study authors will be contacted to request additional clarification. The process of study selection will be reported with a flow diagram [49].

#### Data collection and extraction

We will seek information from individual-participant and/or aggregated data of the eligible studies, covering study identification, study methodology, population, intervention, and outcomes at different time points (see eAppendix-6 for a more detailed list of data items). We will request IPD from the included studies, and we will aim to standardize and harmonize the collected data into a unified format. To ensure data integrity, we will examine for missing, outlier, and duplicated values, assess the adequacy of randomization (if possible with the available data), and cross-check with the summary statistics reported in the published studies. In case of any discrepancies or concerns, we will collaborate with the study authors to address and resolve the issues. When IPD are not available, two independent reviewers will extract aggregated data from the original reports in a Microsoft Access database (see more details in eAppendix-6).

#### Risk-of-*bias* assessment

Two independent reviewers will assess the risk of bias in the eligible studies, specifically focusing on the effects of assignment to the intervention using the Risk of Bias 2 (RoB 2), which considers the domains of the randomization process, deviations from intended interventions, missing outcome data, measurement of the outcome, and selection of reported results [50]. RoB 2 utilizes signaling questions and algorithms to assign domain-level judgements of risk of bias, categorized as “low risk,” “some concerns,” or “high risk” [50]. An overall-level judgement will also be assigned according to the worst judgement in a domain [50]. We will prioritize the information provided by the available IPD over the information available in the original published reports to assess the risk of bias. These judgements will be used to inform the assessment of within-study bias in the evaluation of the confidence of the evidence (see “Confidence in the evidence”).

#### Assessment of applicability

As per our eligibility criteria, the eligible RCTs could provide evidence on the effectiveness of interventions in real-world settings. To further assess potential applicability issues, two independent reviewers will utilize the Rating of Included Trials on the Efficacy-Effectiveness Spectrum (RITES) [51]. The RITES tool considers the domains of participant characteristics, trial setting, flexibility, and clinical relevance of the interventions, with each domain rated on a 5-Likert scale from 1 “strong emphasis on efficacy” to 5 “strong emphasis on effectiveness” [51]. These judgements will be used to inform the assessment of potential indirectness of the evidence (see the “Confidence in the evidence”).

### Data synthesis

#### Effect sizes

The effect size for continuous outcomes will be the standardized mean difference (SMD) due to the use of various rating scales to measure sedation or agitation, for dichotomous outcomes will be the odds ratio (OR) due to its preferable mathematical properties in meta-analysis [52, 53], and for time-to-event outcomes will be the hazard ratios (HRs). To support interpretation of the summary effects, we will aim to transform relative effects to absolute risks using as the control event rate the pooled absolute risk in the reference group (i.e., intramuscular haloperidol) [54]. Effect sizes will be presented along with 95% confidence/credible intervals (95% CI) and 95% prediction intervals (95% PI). Moreover, we will rank the interventions in network meta-analysis using the surface under the cumulative ranking curve (SUCRA) when treatment effects are measured with precision [55, 56].

#### Synthesis approach

We will opt for applying IPD network meta-regression models in a Bayesian framework [57–59], using a random-effects model. The regression models will include independent variables about the intervention, as well as study-level factors and covariates acting as potential prognostic factors and/or effect modifiers, i.e., age, sex assigned at birth, baseline severity of agitation, diagnostic subgroups (e.g., psychosis, alcohol intoxication), medication use before the intervention, setting (psychiatric or general emergency settings and the proportion of patients with alcohol or substance intoxication), route of administration, dose, publication year, and definition of adequate sedation. The final specification of the regression models and potential standardizations of the variables will be determined a posteriori based on the available data across studies and their clinical relevance. A tentative list of potential effect modifiers by order of importance is provided in eAppendix-6.

We will use minimally informative priors for location parameters (i.e., intercept and coefficients) and half-normal distribution for the between-study standard deviations (*τ*). Heterogeneity will be quantified with the between-study variance (*τ*^2^), assumed to be common across the treatment comparisons in the network meta-analysis [47], and the 95% PI of the treatment effects [60].

For missing outcome and/or covariate data, we will consider using multilevel joint modelling multiple imputation by taking into consideration the stratification of patients in trials and the missingness [61, 62]. Multiple imputed datasets will be generated and analyzed, and the results will be combined using Rubin’s rules [63].

Although we aim to obtain IPD from all eligible studies, we anticipate that this may not be the case for all outcomes. In such cases, we will consider a two-stage approach to combine studies with available IPD with those reporting only aggregate data [57, 58, 64] or conventional network meta-analyses based on the aggregated data in a frequentist framework [65, 66], as Bayesian models with IPD integration can be computationally intensive. This decision will be based on the nature of the outcome and the amount of available IPD across studies, allowing a realistic strategy that maintains scientific rigor [25].

#### Transitivity assumption and incoherence

The transitivity assumption is a prerequisite for conducting indirect comparisons and network meta-analysis [47]. We anticipate that the trials fulfilling the eligibility criteria (see the “Eligibility criteria” and eAppendix-3) to be sufficiently similar and that the examined interventions can be jointly randomized. We will also examine distribution of potential effect modifiers among the different treatment comparisons (see in the “Synthesis approach”). The potential statistical disagreement between direct and indirect evidence (incoherence) will be examined for each pairwise comparison using the separating indirect from direct evidence (SIDE) approach [67] and for the entire network using a design-by-treatment interaction test [68].

#### Sensitivity analyses

We will conduct the following sensitivity analysis to investigate the robustness of the findings for the primary outcome: (1) exclusion of open- and single-blinded studies and (2) exclusion of studies with an overall high risk of bias. We will further investigate the potential data availability bias by examining potential differences between studies with available and unavailable IPD in terms of their study design and participant characteristics and their effect sizes.

#### Reporting bias and small-study effects

We will assess reporting biases for each comparison using the Risk Of Bias due to Missing Evidence in Network meta-analysis (ROB-MEN) framework, which considers both within-study and across-study reporting bias [69, 70]. This framework assigns risk levels of “low risk,” “some concerns,” and “high risk” due to missing evidence to each comparison [69, 70]. Small-study effects are examined using contour-enhanced [71] and comparison-adjusted funnel plots [72] and by including the study variance as covariate in a meta-regression model. The assessments will be used to inform reporting biases in the evaluation of the confidence in the evidence.

#### Confidence in the evidence

We will assess confidence in the evidence using the Confidence in Network Meta-Analysis (CINeMA) framework which takes into consideration the domains of within-study bias, indirectness, reporting bias, heterogeneity, imprecision, and incoherence [60, 73]. Specifically, we will assess the confidence in the evidence for the following outcomes: adequate sedation after the intervention (primary outcome), need for additional medication, death, respiratory depression, arrhythmias, oversedation, and at least one extrapyramidal side effect. We will set the margin of equivalence for the odds ratios for these outcomes within the range of 0.83 to 1.20, which is required to assess the domains of imprecision, heterogeneity, and incoherence [60, 73].

#### Statistical software

Data analysis will be conducted in R statistical software [74] using the packages meta [75] and netmeta [65], crossnma [58, 59], and self-programmed routines in JAGS [76, 77].

### Patient and public involvement

The topic of the project was deemed highly relevant by our experiential advisors (consultants or co-authors), consisting of patient and relative representatives from Bündnis für psychisch erkrankte Menschen (BASTA) and Aktionsgemeinschaft der Angehörigen psychisch Kranker (ApK e.V. Bavaria). The experiential advisors will actively participate in meetings regarding the progress of the project and contribute to all important stages of the review such as the design of the protocol and identification of relevant outcomes, the interpretation of findings from an experiential perspective, and the dissemination of findings using accessible language to reach a wider public audience. Patient and public involvement will be reported using the GRIPP2-SF checklist [78].

## Discussion

Severe psychomotor agitation is an emergency condition requiring prompt pharmacological intervention, but no clear evidence-based recommendations exist for choosing among the various options, with current treatment guidelines displaying important inconsistencies [2–9]. To fill this gap and better inform treatment decision-making, our planned individual-participant-data network meta-analysis aims to provide a fine-grained synthesis of the evidence on the comparative effectiveness and tolerability of intramuscular or intravenous drugs administered either as monotherapy or in combination for severe psychomotor agitation in real-world emergency settings.

### Contextualizing with existing literature

Our planned analysis aims to overcome the limitations of previous reviews [18–23]. First, our review aims to provide highly applicable evidence-based information on the effectiveness of pharmacological interventions by designing our eligibility criteria to identify studies conducted in real-world settings, unlike most previous reviews that did not differentiate from explanatory trials [18–22]. This is crucial because explanatory trials, such as those using placebo, conducted by the industry and requiring informed consent before the intervention, typically exclude patients with severe forms of agitation encountered in real-world settings, making their generalizability uncertain [79].

Second, we plan to use an advanced meta-analytic approach, incorporating network meta-analysis and leveraging the advantages of integrating more detailed information from individual-participant data [24, 25]. This approach will enable us to establish hierarchies among the different pharmacological interventions across effectiveness and tolerability outcomes, provide estimates with increased precision, and explore factors that may influence these outcomes, such as differences among diagnostic subgroups and between psychiatric and general emergency settings. Thus, our analysis can extend far beyond a previous network meta-analysis based on aggregated data from studies conducted only in general emergency departments [23]. To our knowledge, such an individual-participant-data network meta-analysis does not exist, highlighting a certain gap in the literature.

### Limitations and challenges

Although an IPD network meta-analysis can offer a more elaborate analysis necessary to provide more precise answers for this topic, it is more complex and time and resource intensive [24, 25, 80–82]. A major challenge will be acquiring IPD, which may take longer than initially planned, and although we will aim and anticipate to acquire most of the IPD, it may still not be feasible to obtain them for some studies or outcomes [42, 83]. We will explore potential data availability biases [84] and consider meta-analytic models allowing the synthesis of studies providing IPD alongside those reporting only aggregate data [57, 58, 64].

Another challenge would be the management, harmonization, and analysis of the IPD datasets, which are expected to vary substantially in format, completeness, and level of detail [80, 81]. Our main goal is to harmonize these datasets into a common format, allowing a detailed synthesis of the evidence. However, decisions regarding the exact model specification and standardization of variables will need to be made a posteriori based on the available data. This process may require trade-offs between preserving data detail and achieving harmonization, but we will make pragmatic choices that maintain scientific rigor.

## Conclusion

Identifying the most appropriate pharmacological intervention for the management of severe psychomotor agitation is crucial in real-world emergency settings, but clear evidence-based recommendations are lacking. We hope the findings of our individual-participant-data network meta-analysis will provide the necessary information on the effectiveness and tolerability of pharmacological interventions to guide treatment decision-making for this common and heterogeneous emergency condition and facilitate the creation of more uniform and acceptable guideline recommendations. Moreover, our analysis will help identify potential gaps in the literature and pharmacological options that may warrant additional research. The findings will be published in a peer-reviewed scientific journal and further disseminated through plain-language summaries and presentations to ensure broad accessibility and uptake, facilitating their implementation in clinical practice.

### Supplementary Information

Additional file 1: eAppendix-1. PRISMA-P. eAppendix-2: Status of the review and PROSPERO registration. eAppendix-3. Table of inclusion and exclusion criteria. eAppendix-4. List of outcomes. eAppendix-5. Search strategies. eAppendix-6. Data items and data extraction of aggregated data

## Data Availability

Not applicable.

## Abbreviations

95% CI: 95% Confidence or credible intervals
95% PI: 95% Prediction intervals
ApK e.V.: Angehörigen psychisch Kranker
BARS: Barnes Akathisia Rating Scale
BASTA: Bündnis für psychisch erkrankte Menschen
CINeMA: Confidence in Network Meta-Analysis
HR: Hazard ratio
IPD: Individual-participant data
MedDRA: Medical Dictionary for Regulatory Activities
OAS: Overt Aggression Scale
OR: Odds ratio
PANSS-EC: Positive and Negative Syndrome Scale-Excitement Component
PRISMA: Preferred Reporting Items for Systematic Reviews and Meta-Analyses
RCT: Randomized controlled trial
RITES: Rating of Included Trials on the Efficacy-Effectiveness Spectrum
ROB-MEN: Risk Of Bias due to Missing Evidence in Network meta-analysis
SAS: Simpson-Angus Scale
SIDE: Separating indirect from direct evidence
SMD: Standardized mean difference
SUCRA: Surface under the cumulative ranking curve
TREC: Tranquilização Rápida-Ensaio Clínico
τ: Between-study standard deviation

## References

[1] APA. Diagnostic and Statistical Manual of Mental Disorders: DSM-5, vol. 5. DC: American psychiatric association Washington; 2013.

[2] Garriga M, Pacchiarotti I, Kasper S, Zeller SL, Allen MH, Vázquez G, Baldaçara L, San L, McAllister-Williams RH, Fountoulakis KN, et al. Assessment and management of agitation in psychiatry: expert consensus. World J Biol Psychiatry. 2016;17(2):86–128. 26912127 10.3109/15622975.2015.1132007

[3] Roppolo LP, Morris DW, Khan F, Downs R, Metzger J, Carder T, Wong AH, Wilson MP. Improving the management of acutely agitated patients in the emergency department through implementation of Project BETA (best practices in the evaluation and treatment of agitation). J Am Coll Emerg Physicians Open. 2020;1(5):898–907. 33145538 10.1002/emp2.12138 PMC7593430

[4] DGPPN: S3-Leitlinie Schizophrenie. In: In*.* Edited by Deutsche Gesellschaft für Psychiatrie und Psychotherapie PuNeVD. 2019.

[5] Pajonk F-G, Messer T, Berzewski H. In: S2k-Leitlinie Notfallpsychiatrie: Springer. 2020.

[6] National Collaborating Centre for Mental H. In: Violence and aggression: short-term management in mental health, health and community settings. 2015. 26180871

[7] Baldaçara L, Diaz AP, Leite P, Pereira LA, Dos Santos RM, Gomes VdP, Calfat EL, Ismael F, Périco CA, Porto DM. Brazilian guidelines for the management of psychomotor agitation. Part 2. Pharmacological approach. Braz J Psychiatry. 2019;41:324–35. 30843960 10.1590/1516-4446-2018-0177 PMC6804299

[8] Brown MD, Byyny R, Diercks DB, Gemme SR, Gerardo CJ, Godwin SA, Hahn SA, Hatten BW, Haukoos JS, Ingalsbe GS. Clinical policy: critical issues in the diagnosis and management of the adult psychiatric patient in the emergency department. Ann Emerg Med. 2017;69(4):480–98. 28335913 10.1016/j.annemergmed.2017.01.036

[9] Galletly C, Castle D, Dark F, Humberstone V, Jablensky A, Killackey E, Kulkarni J, McGorry P, Nielssen O, Tran N. Royal Australian and New Zealand College of Psychiatrists clinical practice guidelines for the management of schizophrenia and related disorders. Aust N Z J Psychiatry. 2016;50(5):410–72. 27106681 10.1177/0004867416641195

[10] TREC Collaborative Group. Rapid tranquillisation for agitated patients in emergency psychiatric rooms: a randomised trial of midazolam versus haloperidol plus promethazine. BMJ. 2003;327(7417):708–13. 14512476 10.1136/bmj.327.7417.708 PMC200800

[11] Alexander J, Tharyan P, Adams C, John T, Mol C, Philip J. Rapid tranquillisation of violent or agitated patients in a psychiatric emergency setting Pragmatic randomised trial of intramuscular lorazepam v haloperidol plus promethazine. Br J Psychiatry. 2004;185:63–9. 15231557 10.1192/bjp.185.1.63

[12] Raveendran NS, Tharyan P, Alexander J, Adams CE. Rapid tranquillisation in psychiatric emergency settings in India: pragmatic randomised controlled trial of intramuscular olanzapine versus intramuscular haloperidol plus promethazine. BMJ. 2007;335(7625):865. 17954514 10.1136/bmj.39341.608519.BE PMC2043469

[13] Huf G, Coutinho ES, Adams CE. Rapid tranquillisation in psychiatric emergency settings in Brazil: pragmatic randomised controlled trial of intramuscular haloperidol versus intramuscular haloperidol plus promethazine. BMJ. 2007;335(7625):869. 17954515 10.1136/bmj.39339.448819.AE PMC2043463

[14] Dib JE, Yaacoub HE, Ikdais WH, Atallah E, Merheb TJ, Ajaltouni J, Akkari M, Mourad M, Nasr ME, Hachem D, et al. Rapid tranquillisation in a psychiatric emergency hospital in Lebanon: TREC-Lebanon - a pragmatic randomised controlled trial of intramuscular haloperidol and promethazine v intramuscular haloperidol, promethazine and chlorpromazine. Psychol Med. 2022;52(13):2751–9. 33402230 10.1017/S0033291720004869

[15] Mantovani C, Labate Cm Fau - Sponholz A, Jr., Sponholz A Jr Fau - de Azevedo Marques JM, de Azevedo Marques Jm Fau - Guapo VG, Guapo Vg Fau - de Simone Brito dos Santos ME, de Simone Brito dos Santos Me Fau - Pazin-Filho A, Pazin-Filho A Fau - Del-Ben CM, Del-Ben CM: Are low doses of antipsychotics effective in the management of psychomotor agitation? A randomized, rated-blind trial of 4 intramuscular interventions. 2013(1533–712X (Electronic)). 10.1097/JCP.0b013e3182900fd6 23609398

[16] Knott JC, Taylor DM, Castle DJ. Randomized clinical trial comparing intravenous midazolam and droperidol for sedation of the acutely agitated patient in the emergency department. Ann Emerg Med. 2006;47(1):61-7. 10.1016/j.annemergmed.2005.07.003. 10.1016/j.annemergmed.2005.07.003 16387219

[17] Chan EW, Lao KSJ, Lam L, Tsui SH, Lui CT, Wong CP, Graham CA, Cheng CH, Chung TS, Lam HF, Ting SM, Knott JC, Taylor DM, Kong DCM, Leung LP, Wong ICK. Intramuscular midazolam, olanzapine, or haloperidol for the management of acute agitation: A multi-centre, double-blind, randomised clinical trial. EClinicalMedicine. 2021;32:100751. 10.1016/j.eclinm.2021.100751. 10.1016/j.eclinm.2021.100751 PMC7910711 33681744

[18] Bak M, Weltens I, Bervoets C, De Fruyt J, Samochowiec J, Fiorillo A, Sampogna G, Bienkowski P, Preuss WU, Misiak B, Frydecka D, Samochowiec A, Bak E, Drukker M, Dom G, The pharmacological management of agitated and aggressive behaviour: A systematic review and meta-analysis. Eur Psychiatry. 2019;57:78-100. 10.1016/j.eurpsy.2019.01.014. 10.1016/j.eurpsy.2019.01.014 30721802

[19] Ostinelli EG, Brooke-Powney MJ, Li X, Adams CE. Haloperidol for psychosis-induced aggression or agitation (rapid tranquillisation). Cochrane Database Syst Rev. 2017;7(7):Cd009377. 28758203 10.1002/14651858.CD009377.pub3 PMC6483410

[20] Zaman H, Sampson SJ, Beck ALS, Sharma T, Clay FJ, Spyridi S, Zhao S, Gillies D. Benzodiazepines for psychosis‐induced aggression or agitation. Cochrane Database Syst Rev 2017;12(12):CD003079. 29219171 10.1002/14651858.CD003079.pub4 PMC6486117

[21] Uribe ES, Bravo-Rodríguez CA, Navarrete-Juárez ME, Medrano-Juarez SB, Lucio RH, Rojas-Guzman KE, Lozano-Carrillo LC: Pharmacological management of acute agitation in psychiatric patients: an umbrella review. 2024. 10.1186/s12888-024-06426-3 PMC11934566 40133850

[22] Muir-Cochrane E, Grimmer K, Gerace A, Bastiampillai T, Oster C, Safety and effectiveness of olanzapine and droperidol for chemical restraint for non-consenting adults: a systematic review and meta-analysis. Australas Emerg Care. 2021;24(2):96-111. 10.1016/j.auec.2020.08.004. 10.1016/j.auec.2020.08.004 33046432

[23] deSouza IS, Thode HC Jr, Shrestha P, Allen R, Koos J, Singer AJ. Rapid tranquilization of the agitated patient in the emergency department: a systematic review and network meta-analysis. Am J Emerg Med. 2022;51:363–73. 34823192 10.1016/j.ajem.2021.11.011

[24] Riley RD, Dias S, Donegan S, Tierney JF, Stewart LA, Efthimiou O, Phillippo DM: Using individual participant data to improve network meta-analysis projects. BMJ Evid Based Med 2022. 10.1136/bmjebm-2022-111931 PMC10313959 35948411

[25] Kanters S, Karim ME, Thorlund K, Anis A, Bansback N. When does the use of individual patient data in network meta-analysis make a difference? A simulation study. BMC Med Res Methodol. 2021;21(1):21. 10.1186/s12874-020-01198-2. 10.1186/s12874-020-01198-2 PMC7805229 33435879

[26] Shamseer L, Moher D, Clarke M, Ghersi D, Liberati A, Petticrew M, Shekelle P, Stewart LA. Preferred Reporting Items for Systematic Review and Meta-Analysis Protocols (PRISMA-P) 2015: elaboration and explanation. BMJ : British Medical Journal. 2015;349:g7647. 10.1136/bmj.g7647 25555855

[27] Dickert NW, Sugarman J. Ethics and regulatory barriers to research in emergency settings. Ann Emerg Med. 2018;72(4):386–8. 30031559 10.1016/j.annemergmed.2018.05.025

[28] Paris G, Bighelli I, Deste G, Siafis S, Schneider-Thoma J, Zhu Y, Davis JM, Vita A, Leucht S. Short-acting intramuscular second-generation antipsychotic drugs for acutely agitated patients with schizophrenia spectrum disorders A systematic review and network meta-analysis. Schizophr Res. 2021;229:3–11. 33607608 10.1016/j.schres.2021.01.021

[29] Chaimani A, Caldwell DM, Li T, Higgins JPT, Salanti G: Undertaking network meta‐analyses. Cochrane handbook for systematic reviews of interventions 2019:285-320.

[30] Gerson R, Malas N, Feuer V, Silver GH, Prasad R, Mroczkowski. Best Practices for Evaluation and Treatment of Agitated Children and Adolescents (BETA) in the Emergency Department: Consensus Statement of the American Association for Emergency Psychiatry. West J Emerg Med. 2019;20(2):409-18. 10.5811/westjem.2019.1.41344. 10.5811/westjem.2019.1.41344 PMC6404720 30881565

[31] Richards JR, Derlet RW, Duncan DR. Chemical restraint for the agitated patient in the emergency department: lorazepam versus droperidol. J Emerg Med. 1998;16(4):567–73. 9696171 10.1016/S0736-4679(98)00045-6

[32] Calver LA, Stokes B, Isbister GK. Sedation assessment tool to score acute behavioural disturbance in the emergency department. Emerg Med Australas. 2011;23(6):732–40. 22151672 10.1111/j.1742-6723.2011.01484.x

[33] Furukawa TA, Akechi T, Wagenpfeil S, Leucht S. Relative indices of treatment effect may be constant across different definitions of response in schizophrenia trials. Schizophr Res. 2011;126(1–3):212–9. 21062670 10.1016/j.schres.2010.10.016

[34] Montoya A, Valladares A, Lizán L, San L, Escobar R, Paz S. Validation of the excited component of the Positive and Negative Syndrome Scale (PANSS-EC) in a naturalistic sample of 278 patients with acute psychosis and agitation in a psychiatric emergency room. Health Qual Life Outcomes. 2011;9:18. 21447155 10.1186/1477-7525-9-18 PMC3078838

[35] Yudofsky SC, Silver JM, Jackson W, Endicott J, Williams D. The Overt Aggression Scale for the objective rating of verbal and physical aggression. Am J Psychiatry. 1986;143:35–9. 3942284 10.1176/ajp.143.1.35

[36] Brown EG, Wood L, Wood S. The Medical Dictionary for Regulatory Activities (MedDRA). Drug Saf. 1999;20(2):109–17. 10082069 10.2165/00002018-199920020-00002

[37] Simpson GM, Angus JWS. A rating scale for extrapyramidal side effects. Acta Psychiatr Scand. 1970;45(S212):11–9. 10.1111/j.1600-0447.1970.tb02066.x 4917967

[38] Barnes TRE. A rating scale for drug-induced akathisia. Br J Psychiatry. 1989;154(5):672–6. 2574607 10.1192/bjp.154.5.672

[39] Bhuiyan PS, Rege NN: ICH harmonised tripartite guideline: guideline for good clinical practice. 1996.

[40] Elbourne DR, Altman DG, Higgins JPT, Curtin F, Worthington HV, Vail A. Meta-analyses involving cross-over trials: methodological issues. Int J Epidemiol. 2002;31(1):140–9. 10.1093/ije/31.1.140. 10.1093/ije/31.1.140 11914310

[41] Donner A, Piaggio G, Villar J. Statistical methods for the meta-analysis of cluster randomization trials. Stat Methods Med Res. 2001;10(5):325–38. 11697225 10.1177/096228020101000502

[42] Veroniki AA, Ashoor HM, Le SPC, Rios P, Stewart LA, Clarke M, Mavridis D, Straus SE, Tricco AC. Retrieval of individual patient data depended on study characteristics: a randomized controlled trial. J Clin Epidemiol. 2019;113:176–88. 31153977 10.1016/j.jclinepi.2019.05.031

[43] Brunoni AR, Tadini L, Fregni F. Changes in clinical trials methodology over time: a systematic review of six decades of research in psychopharmacology. PLoS ONE. 2010;5(3):e9479. 20209133 10.1371/journal.pone.0009479 PMC2831060

[44] Weeks J, Cuthbert A, Alfirevic Z. Trustworthiness assessment as an inclusion criterion for systematic reviews—what is the impact on results? Cochrane Evidence Synthesis and Methods. 2023;1(10):e12037. 10.1002/cesm.12037 PMC11795932 40476011

[45] Weibel S, Popp M, Reis S, Skoetz N, Garner P, Sydenham E. Identifying and managing problematic trials: a research integrity assessment tool for randomized controlled trials in evidence synthesis. Res Synth Methods. 2023;14(3):357–69. 36054583 10.1002/jrsm.1599 PMC10551123

[46] Kataoka Y, Banno M, Tsujimoto Y, Ariie T, Taito S, Suzuki T, Oide S, Furukawa TA. Retracted randomized controlled trials were cited and not corrected in systematic reviews and clinical practice guidelines. J Clin Epidemiol. 2022;150:90–7. 35779825 10.1016/j.jclinepi.2022.06.015

[47] Higgins JPT, Thomas J, Chandler J, Cumpston M, Li T, Page MJ, Welch VA: Cochrane Handbook for Systematic Reviews of Interventions: John Wiley & Sons; 2019. 10.1002/14651858.ED000142 PMC10284251 31643080

[48] Rethlefsen ML, Kirtley S, Waffenschmidt S, Ayala AP, Moher D, Page MJ, Koffel JB, Blunt H, Brigham T, Chang S, et al. PRISMA-S: an extension to the PRISMA statement for reporting literature searches in systematic reviews. Syst Rev. 2021;10(1):39. 10.1186/s13643-020-01542-z. 10.1186/s13643-020-01542-z PMC7839230 33499930

[49] Page MJ, McKenzie JE, Bossuyt PM, Boutron I, Hoffmann TC, Mulrow CD, Shamseer L, Tetzlaff JM, Akl EA, Brennan SE, et al. The PRISMA 2020 statement: an updated guideline for reporting systematic reviews. BMJ. 2021;372:n71. 33782057 10.1136/bmj.n71 PMC8005924

[50] Sterne JAC, Savović J, Page MJ, Elbers RG, Blencowe NS, Boutron I, Cates CJ, Cheng HY, Corbett MS, Eldridge SM, et al. RoB 2: a revised tool for assessing risk of bias in randomised trials. BMJ. 2019;366:l4898. 31462531 10.1136/bmj.l4898

[51] Wieland LS, Berman BM, Altman DG, Barth J, Bouter LM, D’Adamo CR, Linde K, Moher D, Mullins CD, Treweek S, et al. Rating of included trials on the efficacy-effectiveness spectrum: development of a new tool for systematic reviews. J Clin Epidemiol. 2017;84:95–104. 28188898 10.1016/j.jclinepi.2017.01.010 PMC5441969

[52] Doi SA, Furuya-Kanamori L, Xu C, Lin L, Chivese T, Thalib L: Questionable utility of the relative risk in clinical research: a call for change to practice. J Clin Epidemiol 2020. 10.1016/j.jclinepi.2020.08.019 33171273

[53] Bakbergenuly I, Hoaglin DC, Kulinskaya E. Pitfalls of using the risk ratio in meta-analysis. Res Synth Methods. 2019;10(3):398-419. 10.1002/jrsm.1347. 10.1002/jrsm.1347 PMC6767076 30854785

[54] Leucht S, Siafis S, Engel RR, Schneider-Thoma J, Bighelli I, Cipriani A, Furukawa TA, Davis JM. How efficacious are antipsychotic drugs for schizophrenia? An interpretation based on 13 effect size indices. Schizophr Bull. 2022;48(1):27–36. 34405881 10.1093/schbul/sbab094 PMC8781341

[55] Salanti G, Nikolakopoulou A, Efthimiou O, Mavridis D, Egger M, White IR. Introducing the treatment hierarchy question in network meta-analysis. Am J Epidemiol. 2022;191(5):930–8. 35146500 10.1093/aje/kwab278 PMC9071581

[56] Salanti G, Ades AE, Ioannidis JP. Graphical methods and numerical summaries for presenting results from multiple-treatment meta-analysis: an overview and tutorial. J Clin Epidemiol. 2011;64(2):163–71. 20688472 10.1016/j.jclinepi.2010.03.016

[57] Donegan S, Williamson P, D’Alessandro U, Garner P, Smith CT. Combining individual patient data and aggregate data in mixed treatment comparison meta-analysis: individual patient data may be beneficial if only for a subset of trials. Stat Med. 2013;32(6):914–30. 22987606 10.1002/sim.5584

[58] Hamza T, Chalkou K, Pellegrini F, Kuhle J, Benkert P, Lorscheider J, Zecca C, Iglesias-Urrutia CP, Manca A, Furukawa TA, et al. Synthesizing cross-design evidence and cross-format data using network meta-regression. Res Synth Methods. 2023;14:283–300. 36625736 10.1002/jrsm.1619

[59] Hamza T, Schwarzer G, Salanti G: crossnma: cross-design and cross-format synthesis using network meta-analysis and network meta-regression. In*.*; 2022. 10.1186/s12874-023-02130-0 PMC11299362 39103781

[60] Nikolakopoulou A, Higgins JPT, Papakonstantinou T, Chaimani A, Del Giovane C, Egger M, Salanti G. CINeMA: An approach for assessing confidence in the results of a network meta-analysis. PLoS Med, 2020;17(4):e1003082. 10.1371/journal.pmed.1003082 PMC7122720 32243458

[61] Van Buuren S, Groothuis-Oudshoorn K. mice: Multivariate imputation by chained equations in R. J Stat Softw. 2011;45:1–67. 10.18637/jss.v045.i03

[62] Azur MJ, Stuart EA, Frangakis C, Leaf PJ. Multiple imputation by chained equations: what is it and how does it work? Int J Methods Psychiatr Res. 2011;20(1):40–9. 21499542 10.1002/mpr.329 PMC3074241

[63] Rubin DB: Multiple imputation for nonresponse in surveys, vol. 81: John Wiley & Sons; 2004.

[64] Saramago P, Sutton AJ, Cooper NJ, Manca A. Mixed treatment comparisons using aggregate and individual participant level data. Stat Med. 2012;31(28):3516-36. 10.1002/sim.5442. 10.1002/sim.5442 22764016

[65] Balduzzi S, Rücker G, Nikolakopoulou A, Papakonstantinou T, Salanti G, Efthimiou O, Schwarzer G. netmeta: an R package for network meta-analysis using frequentist methods. J Stat Softw. 2023;106(2):1–40.37138589 10.18637/jss.v106.i02

[66] Rücker G: Network meta-analysis, electrical networks and graph theory. 2012(1759–2879 (Print)). 10.1002/jrsm.1058 26053424

[67] König J, Krahn U, Binder H. Visualizing the flow of evidence in network meta-analysis and characterizing mixed treatment comparisons. Stat Med. 2013;32(30):5414-29. 10.1002/sim.6001. 10.1002/sim.6001 24123165

[68] Higgins JP, Jackson D, Barrett JK, Lu G, Ades AE, White IR. Consistency and inconsistency in network meta-analysis: concepts and models for multi-arm studies. Res Synth Methods. 2012;3(2):98–110. 26062084 10.1002/jrsm.1044 PMC4433772

[69] Chiocchia V, Nikolakopoulou A, Higgins JPT, Page MJ, Papakonstantinou T, Cipriani A, Furukawa TA, Siontis GCM, Egger M, Salanti G. ROB-MEN: a tool to assess risk of bias due to missing evidence in network meta-analysis. BMC Med. 2021;19(1):304. 34809639 10.1186/s12916-021-02166-3 PMC8609747

[70] Chiocchia V, Holloway A, Salanti G. Semi-automated assessment of the risk of bias due to missing evidence in network meta-analysis: a guidance paper for the ROB-MEN web-application. BMC Med Res Methodol. 2023;23(1):223. 37805460 10.1186/s12874-023-02038-9 PMC10559514

[71] Peters JL, Sutton AJ, Jones DR, Abrams KR, Rushton L. Contour-enhanced meta-analysis funnel plots help distinguish publication bias from other causes of asymmetry. J Clin Epidemiol. 2008;61(10):991–6. 18538991 10.1016/j.jclinepi.2007.11.010

[72] Chaimani A, Higgins JP, Mavridis D, Spyridonos P, Salanti G. Graphical tools for network meta-analysis in STATA. PLoS ONE. 2013;8(10):e76654. 24098547 10.1371/journal.pone.0076654 PMC3789683

[73] Papakonstantinou T, Nikolakopoulou A, Higgins JPT, Egger M, Salanti G. CINeMA: software for semiautomated assessment of the confidence in the results of network meta-analysis. Campbell Syst Rev. 2020;16(1):e1080. 37131978 10.1002/cl2.1080 PMC8356302

[74] R Core Team. R: a language and environment for statistical computing. 2013.

[75] Balduzzi S, Rücker G, Schwarzer G. How to perform a meta-analysis with R: a practical tutorial. Evid Based Ment Health. 2019;22(4):153–60. 31563865 10.1136/ebmental-2019-300117 PMC10231495

[76] Hornik K, Leisch F, Zeileis A, Plummer M: JAGS: a program for analysis of Bayesian graphical models using Gibbs sampling. In*: 2003 2003*; 2003.

[77] Plummer, M. rjags: Bayesian Graphical Models using MCMC. R package version 4-15, 2023. https://cran.rproject.org/web/packages/rjags/rjags.pdf. Accessed 17 July 2024.

[78] Staniszewska S, Brett J, Simera I, Seers K, Mockford C, Goodlad S, Altman DG, Moher D, Barber R, Denegri S, Entwistle A, Littlejohns P, Morris C, Suleman R, Thomas V, Tysall C, GRIPP2 reporting checklists: tools to improve reporting of patient and public involvement in research. Bmj. 2017;358:j3453. 10.1136/bmj.j3453 PMC5539518 28768629

[79] Chan EW, Taylor DM, Phillips GA, Castle DJ, Knott JC, Kong DCM. May I have your consent? Informed consent in clinical trials — feasibility in emergency situations. J Psychiatr Intensive Care. 2011;7(2):109-13.

[80] Ventresca M, Schünemann HJ, Macbeth F, Clarke M, Thabane L, Griffiths G, Noble S, Garcia D, Marcucci M, Iorio A, et al. Obtaining and managing data sets for individual participant data meta-analysis: scoping review and practical guide. BMC Med Res Methodol. 2020;20(1):113. 32398016 10.1186/s12874-020-00964-6 PMC7218569

[81] Maxwell L, Shreedhar P, Carabali M, Levis B. How to plan and manage an individual participant data meta-analysis. An illustrative toolkit Research Synthesis Methods. 2024;15(1):166–74. 37700398 10.1002/jrsm.1670

[82] Stewart LA, Tierney JF. To IPD or not to IPD? Advantages and disadvantages of systematic reviews using individual patient data. Eval Health Prof. 2002;25(1):76–97. 11868447 10.1177/0163278702025001006

[83] Nevitt SJ, Marson AG, Davie B, Reynolds S, Williams L, Smith CT. Exploring changes over time and characteristics associated with data retrieval across individual participant data meta-analyses: systematic review. BMJ. 2017;357:j1390. 28381561 10.1136/bmj.j1390 PMC5733815

[84] Tsujimoto Y, Fujii T, Onishi A, Omae K, Luo Y, Imai H, Takahashi S, Itaya T, Pinson C, Nevitt SJ. No consistent evidence of data availability bias existed in recent individual participant data meta-analyses: a meta-epidemiological study. J Clin Epidemiol. 2020;118:107–14. 31654789 10.1016/j.jclinepi.2019.10.004

