# Supplementary material for: Effectiveness of pharmacological treatments for severe agitation in real-world emergency settings: protocol of individual-participant-data network meta-analysis
Source: Syst Rev. 2024 Aug 2;13:205. doi: 10.1186/s13643-024-02623-z (PMC11295517; doi:10.1186/s13643-024-02623-z)
Supplement: Supplementary file 1 — Additional file 1: eAppendix-1. PRISMA-P. eAppendix-2: Status of the review and PROSPERO registration. eAppendix-3. Table of inclusion and exclusion criteria. eAppendix-4. List of outcomes. eAppendix-5. Search strategies. eAppendix-6. Data items and data extraction of aggregated data [file 13643_2024_2623_MOESM1_ESM.pdf]

Appendix

Effectiveness of pharmacological treatments for severe agitation in real-world emergency settings: protocol of individual-participant-data network meta-analysis

Table of Contents

eAppendix-1: PRISMA-P 2
eAppendix-2: Status of the review and PROSPERO registration 4
  Status of the review 4
  Modifications from the first version of the PROSPERO protocol 4
eAppendix-3: Table of inclusion and exclusion criteria 6
eAppendix-4: List of outcomes 7
eAppendix-5: Search strategies 9
  BIOSIS Previews 9
  CENTRAL 10
  CINAHL Plus 10
  ClinicalTrials.gov 11
  Embase 12
  LILACS 13
  MEDLINE 14
  ProQuest Dissertations & Theses A&I 14
  PsycINFO 15
  PubMed 16
  WHO ICTRP 17
eAppendix-6: Data items and data extraction of aggregated data 18
  Data items 18
  Methods of data extraction of aggregated data 18
  Tentative list of potential effect-modifiers 19
References 21

The registered PROSPERO protocol is attached in the end of this document.

eAppendix-1: PRISMA-P

PRISMA-P (Preferred Reporting Items for Systematic review and Meta-Analysis Protocols) 2015 checklist: recommended items to address in a systematic review protocol*

Section and topic	Item No	Checklist item	Page
**ADMINISTRATIVE INFORMATION**			
Title:			
Identification	1a	Identify the report as a protocol of a systematic review	1
Update	1b	If the protocol is for an update of a previous systematic review, identify as such	NA
Registration	2	If registered, provide the name of the registry (such as PROSPERO) and registration number	4, 7
Authors:			
Contact	3a	Provide name, institutional affiliation, e-mail address of all protocol authors; provide physical mailing address of corresponding author	1-2
Contributions	3b	Describe contributions of protocol authors and identify the guarantor of the review	23-24
Amendments	4	If the protocol represents an amendment of a previously completed or published protocol, identify as such and list changes; otherwise, state plan for documenting important protocol amendments	Appendix-2
Support:			
Sources	5a	Indicate sources of financial or other support for the review	22
Sponsor	5b	Provide name for the review funder and/or sponsor	22
Role of sponsor or funder	5c	Describe roles of funder(s), sponsor(s), and/or institution(s), if any, in developing the protocol	22
**INTRODUCTION**			
Rationale	6	Describe the rationale for the review in the context of what is already known	5-6
Objectives	7	Provide an explicit statement of the question(s) the review will address with reference to participants, interventions, comparators, and outcomes (PICO)	6
**METHODS**			
Eligibility criteria	8	Specify the study characteristics (such as PICO, study design, setting, time frame) and report characteristics (such as years considered, language, publication status) to be used as criteria for eligibility for the review	7-11, eAppendix-3
Information sources	9	Describe all intended information sources (such as electronic databases, contact with study authors, trial registers or other grey literature sources) with planned dates of coverage	12
Search strategy	10	Present draft of search strategy to be used for at least one electronic database, including planned limits, such that it could be repeated	Appendix-5
Study records:			
Data management	11a	Describe the mechanism(s) that will be used to manage records and data throughout the review	12-13
Selection process	11b	State the process that will be used for selecting studies (such as two independent reviewers) through each phase of the review (that is, screening, eligibility and inclusion in meta-analysis)	12-13
Data collection process	11c	Describe planned method of extracting data from reports (such as piloting forms, done independently, in duplicate), any processes for obtaining and confirming data from investigators	13, eAppendix-6
Data items	12	List and define all variables for which data will be sought (such as PICO items, funding sources), any pre-planned data assumptions and simplifications	7-11, eAppendix-6

Outcomes and prioritization	13	List and define all outcomes for which data will be sought, including prioritization of main and additional outcomes, with rationale	9-10, eAppendix-4
Risk of bias in individual studies	14	Describe anticipated methods for assessing risk of bias of individual studies, including whether this will be done at the outcome or study level, or both; state how this information will be used in data synthesis	13-14
Data synthesis	15a	Describe criteria under which study data will be quantitatively synthesised	15
	15b	If data are appropriate for quantitative synthesis, describe planned summary measures, methods of handling data and methods of combining data from studies, including any planned exploration of consistency (such as I^2, Kendall's τ)	14-16
	15c	Describe any proposed additional analyses (such as sensitivity or subgroup analyses, meta-regression)	16
	15d	If quantitative synthesis is not appropriate, describe the type of summary planned	NA
Meta-bias(es)	16	Specify any planned assessment of meta-bias(es) (such as publication bias across studies, selective reporting within studies)	16
Confidence in cumulative evidence	17	Describe how the strength of the body of evidence will be assessed (such as GRADE)	17

NA: Not applicable.

The copyright for PRISMA-P (including checklist) is held by the PRISMA-P Group and is distributed under a Creative Commons Attribution Licence 4.0.

From: Shamseer L, Moher D, Clarke M, Ghersi D, Liberati A, Petticrew M, Shekelle P, Stewart L, PRISMA-P Group. Preferred reporting items for systematic review and meta-analysis protocols (PRISMA-P) 2015: elaboration and explanation. BMJ. 2015 Jan 2;349(jan02 1):g7647.

eAppendix-2: Status of the review and PROSPERO registration

Status of the review

The initial protocol was registered with PROSPERO on 24.02.2023 prior to conducting the formal search and study selection (ID: CRD42023402365; see the protocol attached in the end of the eAppendix).

At the time of submission, we have conducted the formal search and completed the study selection. We have also contacted all authors of eligible studies. However, we have not yet commenced the formal data extraction and/or harmonization of the IPD.

Modifications from the first version of the PROSPERO protocol

1. The first version of the PROSPERO protocol stated that studies requiring informed consent from patients or legal guardians prior to the study start would be excluded to prevent potential selection bias. However, some of the eligible studies required the presence and/or informed consent of a legal guardian at the study start, e.g., [1], in accordance with their real-world setting of clinical practice. Thus, this requirement is not expected to introduce significant selection bias in these settings. Consequently, we have revised the protocol accordingly, specifying that only studies requiring informed consent from patients before the study start will be excluded (see “Methods”). In a similar vein, we will not exclude studies that lost >10% of the participants because the participants themselves or their legal guardians did not give informed consent after the study start.

2. As we are interested in investigating the effectiveness of intramuscular and intravenous pharmacological interventions in real-world settings, we will evaluate the effectiveness-efficacy spectrum of each study by utilizing the Rating of Included Trials on the Efficacy-Effectiveness Spectrum (RITES) [2].

3. We further clarified in more detail our inclusion criteria. First, studies focusing on patients of advanced age will be excluded due to issues related to drug dosing and increasing likelihood of delirium associated with dementia (which can be a primary cause of agitation in this age group). Second, studies conducted in specialized settings, such as palliative care,

intensive care, and critical care units, will be excluded, our focus lies on general and psychiatric emergency settings.

4. We further clarified in more detail our outcomes. First, we specified the time points for the secondary outcomes. Second, we rephrased our outcome from “time to discharge from the hospital” to “proportion of participants discharged from the hospital or emergency setting” to align with other dichotomous outcomes, as it will not be considered a time-to-event outcome. Third, we added “serious adverse events” as an outcome to encompass all potential serious adverse events, in addition to death, which was already included.

eAppendix-3: Table of inclusion and exclusion criteria

Domain	Inclusion criteria	Exclusion criteria
**Population**	• Adult participants (as defined in the original studies).<br>• Acute and severe psychomotor agitation and/or aggression requiring pharmacological intervention in emergency setting (as defined in the original study).	• Studies focusing on children, adolescents, and patients of advanced age (as defined in the original studies).<br>• Studies focusing on specific reasons of agitation by their inclusion criteria, e.g., dementia or delirium.
**Interventions**	• Intramuscular or intravenous formulations of antipsychotics (e.g., haloperidol, droperidol, olanzapine, aripiprazole, ziprasidone, clotiapine, chlorpromazine, promethazine), benzodiazepines (e.g., lorazepam, midazolam, diazepam), antihistamines (e.g., diphenhydramine), alpha2 adrenergic agonists (e.g., clonidine, dexmedetomidine), ketamine or other drugs used to calm patients with severe agitation, at any dose or their combination.	• Barbiturates.<br>• Studies using oral or inhaled formulations of drugs.
**Comparison groups**	Any of the eligible drug will be compared with each other in a network meta-analysis. Intramuscular haloperidol will serve as the natural comparison group in the presentation of the findings in forest plots.	• Studies that used placebo as comparison group.
**Outcomes**	• Studies focusing on rapid tranquilization by assessing sedation within 30 minutes after the intervention.	• Studies that did not assess sedation within 30 minutes after the intervention.
**Study design and settings**	• Randomised-controlled trials.<br>• Studies conducted in general or psychiatric emergency units.<br>• Studies conducted since 2002.	• Studies conducted in settings requiring informed consent by the patient themselves before the treatment.<br>• Studies conducted in specialized settings, e.g., palliative care, intensive and critical care units.<br>• Studies whose publications have been retracted.<br>• Studies with a high risk of bias in the randomization process (see “Risk of bias assessment”).<br>• Studies with persisting uncertainties regarding eligibility criteria and/or IPD availability due to inadequate author responses, despite our efforts, will be classified as ‘awaiting classification’ and excluded from the analysis.

eAppendix-4: List of outcomes

Outcome	Definition	Type of outcome	Time-point*
Adequate sedation (*primary outcome*)	Any definition used in the original studies; however, if possible, we will try to harmonize the different definitions (e.g., based on clinical judgment or rating scales) to define adequate sedation as "calm" or "asleep," but ideally not oversedated.	Dichotomous outcome.	Primary time-point: within 30 minutes after the first administration (preferably as close as possible to 20 minutes).<br>Secondary time-points: 10 minutes, 30 minutes, 45 minutes, 60 minutes, 2 hours, 4 hours, 24 hours after the first administration of the intervention.
Time to adequate sedation		Time-to-event outcome.	Not applicable.
Use of additional pharmacological treatment for tranquilization	Any definition used in the original studies including the use of an additional dose of the same drug or the use of another drug to achieve tranquilization.	Dichotomous outcome.	30 minutes, 1 hour, 2 hours, 4 hours, and 24 hours.
Use of physical restrains	Any definition used in the original studies.	Dichotomous outcome.	
Severity of agitation or aggression	Any rating scale used to measure agitated or aggressive behavior such as the Positive and Negative Syndrome Scale Excitement Component (PANSS-EC) [3] and Overt Aggression Scale (OAS) [4]. Other relevant scales will be eligible if they have been used in the original studies. Given that various rating scales are expected to be used across studies, we will use the standardized mean difference as the effect size.	Continuous outcome.	
Discharge from the hospital and/or the emergency unit.	Any definition used in the original studies.	Dichotomous outcome.	
Adverse events	• Death due to any cause.<br>• Serious adverse event as defined by the ICH guidelines [5].<br>• Seizure.<br>• Dystonia.<br>• Akathisia.<br>• Parkinsonism.<br>• Use of antiparkinsonian medications as a proxy for extrapyramidal side-effects.<br>• QTc interval prolongation.<br>• Arrhythmia.<br>• Fall.<br>• Respiratory distress.<br>• Aspiration.<br>• Allergic reaction.<br>• Bronchospasm.<br>• Oversedation.<br>• Hypotension.<br>• Nausea.<br>• Vomiting.	Dichotomous outcomes.	

	We will aim to harmonize different terms for specific adverse events such as by using the MedDRA terminology [6]; and possibly pool data from similar adverse events across studies, if clinically relevant, to allow a comprehensive synthesis of the evidence.		
Severity of extrapyramidal side-effects	Any rating scale to measure drug-induced movement disorders, including scales for i) extrapyramidal side-effects e.g., Simpson Agnus Scale (SAS) [7], ii) akathisia, e.g., as Barnes Akathisia Rating Scale (BARS) [8], and dyskinesia, e.g., Abnormal Involuntary Movement Scale [9]. Other relevant scales will be eligible if they have been used in the original studies. If various rating scales are expected to be used across studies, we will use the standardized mean difference as the effect size.	Continuous outcomes.	
Dropouts	• Dropout due to any reason.<br>• Dropout due to ineffectiveness.<br>• Dropout due to adverse events.	Dichotomous outcomes.	

*It is expected that outcomes, especially secondary outcomes, will be measured at different time points across the studies. In that case, we will aim to pool data from different time points across studies, if possible and clinically relevant, to allow a comprehensive synthesis of the evidence.

eAppendix-5: Search strategies

The search strategies will be developed in collaboration with Dr. Farhad Shokraneh, an expert information specialist (see “Acknowledgements”). The search will be conducted by adhering to the Cochrane Handbook for Systematic Review of Interventions [10] and Cochrane’s MECIR [11].

Keywords will be selected through expert opinions (co-authors of the protocol with expertise in systematic reviews and clinical trials), previous literature reviews, controlled vocabulary (CINAHL Headings, Medical Subject Headings, and Excerpta Medica Tree), and by reviewing primary search results. We will use keywords for agitation, tranquilization treatments, emergency settings. We will also use validated search filters to retrieve randomized controlled trials from CINAHL [12], Embase [13], MEDLINE [10], and PubMed [10]. Since there is no validated search filter for PsycINFO, we will use a search strategy adapted from CADTH [14]. The databases will be searched from database inception without restrictions in terms of publication status, document type and language.

The search strategies for BIOSIS, CENTRAL, CINAHL Plus, clinicaltrials.gov, EMBASE, LILACS, MEDLINE via Ovid, ProQuest, PsycINFO, and PubMed are reported below.

BIOSIS Previews

Web of Science Search Strategy (v0.1)

Database: BIOSIS Previews

Entitlements:

- BIOSIS.PREVIEWS:

Search: Aggression* OR Aggressive Attitude* OR Aggressive Behavior* OR Aggressive Behaviour* OR Aggressive Reaction* OR Aggressiveness* OR Aggressivity OR Agitat* OR Dangerous Behavior* OR Dangerous Behaviour* OR Psychomotor Excitement* OR Psychomotor Hyperactivit* OR Psychomotor Restless* OR Violen* (Topic) AND Emergency or Emergencies or Intensive Care Unit* or ICU or ICUs or Emergicenter* or Acute Psychiatric or Acute Mental or Acute Behavio* or Mental Hospital* or Mental Institut* or Psychiatric Clinic*

or Psychiatric Hospital* or Psychiatric Institut* or Psychiatric Ward* or Psychiatric Unit* (Topic) AND Parenteral* OR Intramuscular* OR Intra-Muscular* OR "IM" OR Intravenous* OR Intra-Venous* OR "IV" OR Tranquil* (Topic)

CENTRAL

Advanced Search/Search Manager

([mh ^Aggression] OR [mh ^"Psychomotor Agitation"] OR [mh ^Violence] OR (Aggression* OR Aggressive Attitude* OR Aggressive Behavior* OR Aggressive Behaviour* OR Aggressive Reaction* OR Aggressiveness* OR Aggressivity OR Agitat* OR Dangerous Behavior* OR Dangerous Behaviour* OR Psychomotor Excitement* OR Psychomotor Hyperactivit* OR Psychomotor Restless* OR Violen*):ti,ab) AND ([mh ^"Injections, Intramuscular"] OR [mh ^"Injections, Intravenous"] OR [mh ^"Administration, Intravenous"] OR [mh ^"Tranquilizing Agents"] OR (Parenteral* OR Intramuscular* OR Intra-Muscular* OR IM OR Intravenous* OR Intra-Venous* OR IV OR Tranquil*):ti,ab) AND ([mh ^Emergencies] OR [mh ^"Emergency Medicine"] OR [mh ^"Emergency Service, Hospital"] OR [mh ^"Emergency Services, Psychiatric"] OR [mh ^"Emergency Treatment"] OR [mh ^"Hospitals, Psychiatric"] OR [mh ^"Emergency Medical Services"] OR [mh ^"Evidence-Based Emergency Medicine"] OR (Emergency OR Emergencies OR Intensive Care Unit* OR ICU OR ICUs OR Emergicenter* OR Acute Psychiatric OR Acute Mental OR Acute Behavio* OR Mental Hospital* OR Mental Institut* OR Psychiatric Clinic* OR Psychiatric Hospital* OR Psychiatric Institut* OR Psychiatric Ward* OR Psychiatric Unit*):ti,ab OR Emergency:kw) in Trials

CINAHL Plus

(((MH "Aggression") OR (MH "Violence") OR (MH "Agitation") OR (MH "Psychomotor Agitation")) OR TI (Aggression* or Aggressive Attitude* or Aggressive Behavior* or Aggressive Behaviour* or Aggressive Reaction* or Aggressiveness* or Aggressivity or Agitat* or Dangerous Behavior* or Dangerous Behaviour* or Psychomotor Excitement* or Psychomotor Hyperactivit* or Psychomotor Restless* or Violen*) OR AB (Aggression* or Aggressive Attitude* or Aggressive Behavior* or Aggressive Behaviour* or Aggressive

Reaction* or Aggressiveness* or Aggressivity or Agitat* or Dangerous Behavior* or Dangerous Behaviour* or Psychomotor Excitement* or Psychomotor Hyperactivit* or Psychomotor Restless* or Violen*)) AND (((MH "Emergencies") OR (MH "Psychiatric Emergencies") OR (MH "Emergency Service") OR (MH "Emergency Services, Psychiatric") OR (MH "Emergency Patients") OR (MH "Emergency Medicine") OR (MH "Emergency Medical Services") OR (MH "Emergency Treatment") OR (MH "Hospitals, Psychiatric")) OR TI (Emergency or Emergencies or Intensive Care Unit* or ICU or ICUs or Emergicenter* or Acute Psychiatric or Acute Mental or Acute Behavio* or Mental Hospital* or Mental Institut* or Psychiatric Clinic* or Psychiatric Hospital* or Psychiatric Institut* or Psychiatric Ward* or Psychiatric Unit*) OR AB (Emergency or Emergencies or Intensive Care Unit* or ICU or ICUs or Emergicenter* or Acute Psychiatric or Acute Mental or Acute Behavio* or Mental Hospital* or Mental Institut* or Psychiatric Clinic* or Psychiatric Hospital* or Psychiatric Institut* or Psychiatric Ward* or Psychiatric Unit*)) AND (((MH "Injections, Intramuscular") OR (MH "Injections, Intravenous") OR (MH "Tranquilizing Agents")) OR TI (Parenteral* OR Intramuscular* OR Intra-Muscular* OR "IM" OR Intravenous* OR Intra-Venous* OR "IV" OR Tranquil*) OR AB (Parenteral* OR Intramuscular* OR Intra-Muscular* OR "IM" OR Intravenous* OR Intra-Venous* OR "IV" OR Tranquil*)) AND ((MH ("Randomized Controlled Trials" OR "Double-Blind Studies" OR "Single-Blind Studies" OR "Random Assignment" OR "Pretest-Posttest Design" OR "Cluster Sample" OR "Placebos" OR "Crossover Design" OR "Comparative Studies") OR TI (Randomised OR Randomized OR Trial) OR AB (Random* OR (Control W5 Group) OR (Cluster W3 RCT)) OR (MH ("Sample Size") AND AB (Assigned OR Allocated OR Control)) OR PT (Randomized Controlled Trial)) NOT ((MH Animals+ OR MH (Animal Studies) OR TI (Animal Model*)) NOT MH (Human)))

Limiters - Exclude MEDLINE records

ClinicalTrials.gov

Advanced Search

Condition or disease: Aggression OR Aggressive OR Agitation OR Agitated OR Violence

Study type: Interventional Studies (Clinical Trials)

Other terms: Randomized

Embase

#1 Randomized controlled trial/ or Controlled clinical study/ or randomization/ or intermethod comparison/ or double blind procedure/ or human experiment/ or (random$ or placebo or (open adj label) or ((double or single or doubly or singly) adj (blind or blinded or blindly)) or parallel group$1 or crossover or cross over or ((assign$ or match or matched or allocation) adj5 (alternate or group$1 or intervention$1 or patient$1 or subject$1 or participant$1)) or assigned or allocated or (controlled adj7 (study or design or trial)) or volunteer or volunteers).ti,ab. or (compare or compared or comparison or trial).ti. or ((evaluated or evaluate or evaluating or assessed or assess) and (compare or compared or comparing or comparison)).ab.

#2 (random$ adj sampl$ adj7 ("cross section$" or questionnaire$1 or survey$ or database$1)).ti,ab. not (comparative study/ or controlled study/ or randomi?ed controlled.ti,ab. or randomly assigned.ti,ab.)

#3 Cross-sectional study/ not (randomized controlled trial/ or controlled clinical study/ or controlled study/ or (randomi?ed controlled or control group$1).ti,ab.)

#4 (((case adj control$) and random$) not randomi?ed controlled).ti,ab.

#5 (Systematic review not (trial or study)).ti.

#6 (nonrandom$ not random$).ti,ab.

#7 ("Random field$" or (random cluster adj3 sampl$)).ti,ab.

#8 (review.ab. and review.pt.) not trial.ti.

#9 "we searched".ab. and (review.ti. or review.pt.)

#10 ("update review" or (databases adj4 searched)).ab.

#11 (rat or rats or mouse or mice or swine or porcine or murine or sheep or lambs or pigs or piglets or rabbit or rabbits or cat or cats or dog or dogs or cattle or bovine or monkey or monkeys or trout or marmoset$1).ti. and animal experiment/

#12 Animal experiment/ not (human experiment/ or human/)

#13 or/2-12

#14 1 not 13

#15 Aggression/ or Aggressiveness/ or Agitation/ or Violence/ or (Aggression* or Aggressive Attitude* or Aggressive Behavior* or Aggressive Behaviour* or Aggressive Reaction* or Aggressiveness* or Aggressivity or Agitat* or Dangerous Behavior* or Dangerous Behaviour* or Psychomotor Excitement* or Psychomotor Hyperactivit* or Psychomotor Restless* or Violen*).ti,ab.

#16 Parenteral Drug Administration/ or Intravascular Drug Administration/ or Intravenous Drug Administration/ or Intramuscular Drug Administration/ or Tranquilizer/ or (Parenteral* or Intramuscular* or Intra-Muscular* or IM or Intravenous* or Intra-Venous* or IV or Tranquil*).ti,ab.

#17 Emergency/ or Psychiatric Emergency/ or "Agents Used in Emergency Medicine"/ or Emergency Treatment/ or Psychiatric Emergency Service/ or Psychiatric Emergency/ or Hospital Emergency Service/ or Emergency Health Service/ or Emergency Ward/ or Emergency Medicine/ or Emergency Management/ or Evidence Based Emergency Medicine/ or Emergency Care/ or Mental Hospital/ or Emergency.af. or (Emergency or Emergencies or Intensive Care Unit* or ICU or ICUs or Emergicenter* or Acute Psychiatric or Acute Mental or Acute Behavio* or Mental Hospital* or Mental Institut* or Psychiatric Clinic* or Psychiatric Hospital* or Psychiatric Institut* or Psychiatric Ward* or Psychiatric Unit*).ti,ab.

#18 14 and 15 and 16 and 17

LILACS

(Aggression* OR Aggressive Attitude* OR Aggressive Behavior* OR Aggressive Behaviour* OR Aggressive Reaction* OR Aggressiveness* OR Aggressivity OR Agitat* OR Dangerous Behavior* OR Dangerous Behaviour* OR Psychomotor Excitement* OR Psychomotor Hyperactivit* OR Psychomotor Restless* OR Violen*) AND (Emergency OR Emergencies OR Intensive Care Unit* OR ICU OR ICUs OR Emergicenter* OR Acute Psychiatric OR Acute

Mental OR Acute Behavio* OR Mental Hospital* OR Mental Institut* OR Psychiatric Clinic* OR Psychiatric Hospital* OR Psychiatric Institut* OR Psychiatric Ward* OR Psychiatric Unit*) AND (Parenteral* OR Intramuscular* OR Intra-Muscular* OR "IM" OR Intravenous* OR Intra-Venous* OR "IV" OR Tranquil*)

MEDLINE

Database: Ovid MEDLINE(R) ALL

#1 Aggression/ or Psychomotor Agitation/ or Violence/ or (Aggression* or Aggressive Attitude* or Aggressive Behavior* or Aggressive Behaviour* or Aggressive Reaction* or Aggressiveness* or Aggressivity or Agitat* or Dangerous Behavior* or Dangerous Behaviour* or Psychomotor Excitement* or Psychomotor Hyperactivit* or Psychomotor Restless* or Violen*).ti,ab.

#2 Injections, Intramuscular/ or Injections, Intravenous/ or Administration, Intravenous/ or Tranquilizing Agents/ or (Parenteral* or Intramuscular* or Intra-Muscular* or IM or Intravenous* or Intra-Venous* or IV or Tranquil*).ti,ab.

#3 Emergencies/ or Emergency Medicine/ or Emergency Service, Hospital/ or Emergency Services, Psychiatric/ or Emergency Treatment/ or Hospitals, Psychiatric/ or Emergency Medical Services/ or Evidence-Based Emergency Medicine/ or Emergency.af. or (Emergency or Emergencies or Intensive Care Unit* or ICU or ICUs or Emergicenter* or Acute Psychiatric or Acute Mental or Acute Behavio* or Mental Hospital* or Mental Institut* or Psychiatric Clinic* or Psychiatric Hospital* or Psychiatric Institut* or Psychiatric Ward* or Psychiatric Unit*).ti,ab.

#4 ((Randomized Controlled Trial or Controlled Clinical Trial).pt. or (Randomi?ed or Placebo or Randomly or Trial or Groups).ab. or Drug Therapy.fs.) not (exp Animals/ not Humans.sh.)

#5 1 and 2 and 3 and 4

ProQuest Dissertations & Theses A&I

(title(Aggression* OR Aggressive Attitude* OR Aggressive Behavior* OR Aggressive Behaviour* OR Aggressive Reaction* OR Aggressiveness* OR Aggressivity OR Agitat* OR Dangerous Behavior* OR Dangerous Behaviour* OR Psychomotor Excitement* OR

Psychomotor Hyperactivit* OR Psychomotor Restless* OR Violen*) OR abstract(Aggression* OR Aggressive Attitude* OR Aggressive Behavior* OR Aggressive Behaviour* OR Aggressive Reaction* OR Aggressiveness* OR Aggressivity OR Agitat* OR Dangerous Behavior* OR Dangerous Behaviour* OR Psychomotor Excitement* OR Psychomotor Hyperactivit* OR Psychomotor Restless* OR Violen*)) AND (title(Emergency OR Emergencies OR Intensive Care Unit* OR ICU OR ICUs OR Emergicenter* OR Acute Psychiatric OR Acute Mental OR Acute Behavio* OR Mental Hospital* OR Mental Institut* OR Psychiatric Clinic* OR Psychiatric Hospital* OR Psychiatric Institut* OR Psychiatric Ward* OR Psychiatric Unit*) OR abstract(Emergency OR Emergencies OR Intensive Care Unit* OR ICU OR ICUs OR Emergicenter* OR Acute Psychiatric OR Acute Mental OR Acute Behavio* OR Mental Hospital* OR Mental Institut* OR Psychiatric Clinic* OR Psychiatric Hospital* OR Psychiatric Institut* OR Psychiatric Ward* OR Psychiatric Unit*)) AND (title(Parenteral* OR Intramuscular* OR Intra-Muscular* OR "IM" OR Intravenous* OR Intra-Venous* OR "IV" OR Tranquil*) OR abstract(Parenteral* OR Intramuscular* OR Intra-Muscular* OR "IM" OR Intravenous* OR Intra-Venous* OR "IV" OR Tranquil*))

PsycINFO

Database: APA PsycInfo

#1 Aggressive Behavior/ or Aggressiveness/ or Agitation/ or Violence/ or Patient Violence/ or (Aggression* or Aggressive Attitude* or Aggressive Behavior* or Aggressive Behaviour* or Aggressive Reaction* or Aggressiveness* or Aggressivity or Agitat* or Dangerous Behavior* or Dangerous Behaviour* or Psychomotor Excitement* or Psychomotor Hyperactivit* or Psychomotor Restless* or Violen*).ti,ab.

#2 Intramuscular Injections/ or Intravenous Injections/ or Tranquilizing Drugs/ or (Parenteral* or Intramuscular* or Intra-Muscular* or IM or Intravenous* or Intra-Venous* or IV or Tranquil*).ti,ab.

#3 Emergency Management/ or Emergency Medicine/ or Emergency Services/ or Psychiatric Hospitals/ or Psychiatric Units/ or Emergency.af. or (Emergency or Emergencies or Intensive

Care Unit* or ICU or ICUs or Emergicenter* or Acute Psychiatric or Acute Mental or Acute Behavio* or Mental Hospital* or Mental Institut* or Psychiatric Clinic* or Psychiatric Hospital* or Psychiatric Institut* or Psychiatric Ward* or Psychiatric Unit*).ti,ab.

#4 exp Clinical Trials/ or Placebo/ or (random* or sham or placebo* or ((singl* or doubl*) adj (blind* or dumm* or mask*)) or ((tripl* or trebl*) adj (blind* or dumm* or mask*)) or (control* adj3 (study or studies or trial* or group*)) or Nonrandom* or non random* or non-random* or quasi-random* or quasirandom* or allocated or ((open label or open-label) adj5 (study or studies or trial*)) or ((equivalence or superiority or non-inferiority or noninferiority) adj3 (study or studies or trial*)) or ((pragmatic or practical) adj3 trial*) or ((quasiexperimental or quasi-experimental) adj3 (study or studies or trial*)) or (phase adj3 (III or "3") adj3 (study or studies or trial*))).ti,ab,hw.

#5 1 and 2 and 3 and 4

PubMed

(Aggression[MH:NoExp] OR "Psychomotor Agitation"[MH:NoExp] OR Violence[MH:NoExp] OR Aggression*[TIAB] OR Aggressive Attitude*[TIAB] OR Aggressive Behavior*[TIAB] OR Aggressive Behaviour*[TIAB] OR Aggressive Reaction*[TIAB] OR Aggressiveness*[TIAB] OR Aggressivity[TIAB] OR Agitat*[TIAB] OR Dangerous Behavior*[TIAB] OR Dangerous Behaviour*[TIAB] OR Psychomotor Excitement*[TIAB] OR Psychomotor Hyperactivit*[TIAB] OR Psychomotor Restless*[TIAB] OR Violen*[TIAB]) AND ("Injections, Intramuscular"[MH:NoExp] OR "Injections, Intravenous"[MH:NoExp] OR "Administration, Intravenous"[MH:NoExp] OR "Tranquilizing Agents"[MH:NoExp] OR Parenteral*[TIAB] OR Intramuscular*[TIAB] OR Intra-Muscular*[TIAB] OR IM[TIAB] OR Intravenous*[TIAB] OR Intra-Venous*[TIAB] OR IV[TIAB] OR Tranquil*[TIAB]) AND (Emergencies[MH:NoExp] OR "Emergency Medicine"[MH:NoExp] OR "Emergency Service, Hospital"[MH:NoExp] OR "Emergency Services, Psychiatric"[MH:NoExp] OR "Emergency Treatment"[MH:NoExp] OR "Hospitals, Psychiatric"[MH:NoExp] OR "Emergency Medical Services"[MH:NoExp] OR "Evidence-Based Emergency Medicine"[MH:NoExp] OR Emergency[All Fields] OR

Emergencies[TIAB] OR Intensive Care Unit*[TIAB] OR ICU[TIAB] OR ICUs[TIAB] OR Emergicenter*[TIAB] OR Acute Psychiatric[TIAB] OR Acute Mental[TIAB] OR Acute Behavio*[TIAB] OR Mental Hospital*[TIAB] OR Mental Institut*[TIAB] OR Psychiatric Clinic*[TIAB] OR Psychiatric Hospital*[TIAB] OR Psychiatric Institut*[TIAB] OR Psychiatric Ward*[TIAB] OR Psychiatric Unit*[TIAB]) AND ((Randomized Controlled Trial[PT] OR Controlled Clinical Trial[PT] OR Pragmatic Clinical Trial[PT] OR Randomized[TIAB] OR Randomised[TIAB] OR Placebo[TIAB] OR Randomly[TIAB] OR Trial[TIAB] OR Groups[TIAB]) NOT (Animals[MH] NOT Humans[MH]))

WHO ICTRP

(Aggression OR Aggressive Behavior OR Aggressive Reaction OR Aggressiveness OR Aggressivity OR Agitation OR Dangerous Behavior OR Psychomotor Excitement OR Psychomotor Hyperactivity OR Psychomotor Restlessness OR Violence) AND (Randomized OR Randomised)

eAppendix-6: Data items and data extraction of aggregated data

Data items

We will seek information from individual-participant and/or aggregated data for the following data items:

- Study identifiers: Name of the first author, publication year, trial registration identifier.
- Study methodology: Inclusion and exclusion criteria of the study, number of arms, number of medications, sample size, country, number of sites, sponsorship, setting (e.g., psychiatric or general emergency department), proportion of patients with alcohol or substance intoxication), and necessary information about the assessment of risk of bias using RoB-2 [15] and applicability using the RITES tool [2].
- Population characteristics: Definition and severity of agitation, age, sex assigned at birth, weight, diagnostic subgroup underlying the agitation and criteria used, medical and psychiatric comorbidities, and medication use before the intervention.
- Intervention characteristics: Name of the drugs, dose, route of administration, co-interventions.
- Outcomes at different timepoints (see "Outcomes" and eAppendix-4).

These data items are crucial for characterizing the sample, assessing the risk of bias, and for the synthesis approach, including exploring heterogeneity. We will consider additional variables if identified in the eligible trials and deemed clinically relevant. Moreover, we present below a tentative list of potential effect-modifiers (by order of importance).

Methods of data extraction of aggregated data

When the individual-participant-data are not available for eligible trials (e.g., for certain outcomes), we will extract aggregated data from original reports. At least two independent reviewers will conduct the data extraction and enter the into a Microsoft Access database that allows the automatic identification of discrepancies between the two reviewers, as used in our previous studies [16, 17]. Any unresolved discrepancies between the two reviewers will be

reconciled with discussion with a third more senior reviewer, or by contacting the study authors for additional information.

For continuous measures, we will extract the mean, standard deviation and the number of participants that these summary data would refer for each of the study arms. We will prefer extracting results from all randomized participants, based on appropriate imputation or modeling methods to handle missing outcome data such as mixed models for repeated measures (MMRM) and multiple imputations, over the last observation carried forward (LOCF) or data from completers only. We will also prefer extracting change over endpoint scores, but both would be eligible and combined using standardized mean differences (see "Effect sizes")[18]. Missing standard deviations will be derived according to the following hierarchy from: standard errors, test statistics (e.g., t- or p-values)[10], by contacting the study authors for additional information, or by using a validated imputation [19].

For dichotomous measures, we will extract the number of participants having the event and the total number of participants randomized to each of the study arms. We will prefer results from all randomized participants or appropriate imputation/modeling methods to handle missing data, as mentioned for continuous measures above, but if they are not available, we will assume that participants lost to follow-up did not have the event. Nonetheless, we do not anticipate a substantial number of dropouts in these short-duration trials. This would be a conservative assumption for effectiveness outcomes and a reasonable one for adverse events given the short duration of the trials. We will also extract data from Kaplan-Meier curves in the case of time-to-event outcomes according to the Cochrane Handbook [10].

Tentative list of potential effect-modifiers

There are insufficient data on potential factors that could act as effect modifiers for the effects of pharmacological interventions for agitation [20, 21], and our work will integrate detailed information from individual-participant data to fill this gap.

From the list of the factors/covariates reported in the "Synthesis Approach", we provide below a tentative list of potential effect-modifiers that we will examine in order of their potential importance:

1. Diagnostic subgroups: These may show differences in the effectiveness of various pharmacological options and are used to guide treatment choices in some recommendations [22, 23].
2. Dose of the drug: The dose can be an important effect-modifier, and we aim to explore potential dose-effect relationships. However, the exact methodology and any potential standardization of the doses across different interventions cannot be predefined due to the various pharmacological options (e.g., different compounds, monotherapy or combination treatment, intramuscular or intravenous administration, see also "Interventions"). Any decisions about the dose will be taken *a posteriori* based on the available data across studies and the clinical relevance.
3. Baseline severity of agitation.
4. Setting: There may be potential differences between general and psychiatric emergency departments.
5. Use of medication before the injection.
6. Age.
7. Sex assigned at birth.

We will consider additional variables if they are identified in the eligible trials and are deemed important potential effect-modifiers based on their clinical relevance and/or in the meta-regression analysis. For example, we will also examine the potential influence of publication year and the definition of adequate sedation on the outcomes.

As mentioned in the "Synthesis approach", the final specification of the regression models and potential standardizations of the variables will be determined *a posteriori* based on the available data across studies and their clinical relevance.

References

1. Alexander J, Tharyan P, Adams C, John T, Mol C, Philip J: **Rapid tranquillisation of violent or agitated patients in a psychiatric emergency setting. Pragmatic randomised trial of intramuscular lorazepam v. haloperidol plus promethazine**. *Br J Psychiatry* 2004, **185**:63-69.
2. Wieland LS, Berman BM, Altman DG, Barth J, Bouter LM, D'Adamo CR, Linde K, Moher D, Mullins CD, Treweek S *et al*: **Rating of Included Trials on the Efficacy-Effectiveness Spectrum: development of a new tool for systematic reviews**. *J Clin Epidemiol* 2017, **84**:95-104.
3. Montoya A, Valladares A, Lizán L, San L, Escobar R, Paz S: **Validation of the Excited Component of the Positive and Negative Syndrome Scale (PANSS-EC) in a naturalistic sample of 278 patients with acute psychosis and agitation in a psychiatric emergency room**. *Health Qual Life Outcomes* 2011, **9**:18.
4. Yudofsky SC, Silver JM, Jackson W, Endicott J, Williams D: **The Overt Aggression Scale for the objective rating of verbal and physical aggression**. *The American journal of psychiatry* 1986.
5. Bhuiyan PS, Rege NN: **ICH harmonised tripartite guideline: guideline for good clinical practice**. 1996.
6. Brown EG, Wood L, Wood S: **The medical dictionary for regulatory activities (MedDRA)**. *Drug Saf* 1999, **20**(2):109-117.
7. Simpson GM, Angus JWS: **A rating scale for extrapyramidal side effects**. *Acta Psychiatrica Scandinavica* 1970, **45**(S212):11-19.
8. Barnes TRE: **A rating scale for drug-induced akathisia**. *The British Journal of Psychiatry* 1989, **154**(5):672-676.
9. Guy W: **ECDEU assessment manual for psychopharmacology**: US Department of Health, Education, and Welfare, Public Health Service …; 1976.
10. Higgins JPT, Thomas J, Chandler J, Cumpston M, Li T, Page MJ, Welch VA: **Cochrane handbook for systematic reviews of interventions**: John Wiley & Sons; 2019.
11. Higgins JPT, Lasserson T, Chandler J, Tovey D, Thomas J, Flemyng ECR: **Methodological expectations of cochrane intervention reviews (MECIR). 2022**. In.; 2023.
12. Glanville J, Dooley G, Wisniewski S, Foxlee R, Noel-Storr A: **Development of a search filter to identify reports of controlled clinical trials within CINAHL Plus**. *Health Information & Libraries Journal* 2019, **36**(1):73-90.
13. Glanville J, Foxlee R, Wisniewski S, Noel-Storr A, Edwards M, Dooley G: **Translating the Cochrane EMBASE RCT filter from the Ovid interface to Embase. com: a case study**. *Health Information & Libraries Journal* 2019, **36**(3):264-277.
14. John W Scott Health Sciences Library: **Randomized Controlled Trials / Controlled Clinical Trials: A Cut and Paste Search Strategy adapted from CADTH for Ovid PsycINFO. Adapted from: "Canadian Agency for Drugs and Technology in Health [Internet]. Strings attached: CADTH's database search filters - randomized controlled trials / controlled clinical trials — OVID Medline, Embase, PsycINFO. Ottawa: CADTH; 2018." John W. Scott Health Sciences Library, University of Alberta Rev. December 9, 2020. Available from: https://docs.google.com/document/d/1g7vXZz2CAAqZpbHoOFxxiCVDdCW6bRsDuvqYZDiMH5s/edit.** .
15. Sterne JAC, Savović J, Page MJ, Elbers RG, Blencowe NS, Boutron I, Cates CJ, Cheng HY, Corbett MS, Eldridge SM *et al*: **RoB 2: a revised tool for assessing risk of bias in randomised trials**. *Bmj* 2019, **366**:l4898.
16. Siafis S, Schneider-Thoma J, Hamza T, Bighelli I, Dong S, Hansen WP, Davis JM, Salanti G, Leucht S: **Efficacy of clozapine compared with other second-generation antipsychotic drugs in patients with treatment-resistant schizophrenia: protocol**

for a systematic review and individual patient data meta-analysis of randomised controlled trials. *BMJ Open* 2023, **13**(2):e064504.
17. Huhn M, Nikolakopoulou A, Schneider-Thoma J, Krause M, Samara M, Peter N, Arndt T, Bäckers L, Rothe P, Cipriani A *et al*: **Comparative efficacy and tolerability of 32 oral antipsychotics for the acute treatment of adults with multi-episode schizophrenia: a systematic review and network meta-analysis**. *Lancet* 2019, **394**(10202):939-951.
18. Ostinelli EA-O, Efthimiou OA-O, Luo YA-O, Miguel CA-O, Karyotaki EA-O, Cuijpers PA-O, Furukawa TA-O, Salanti GA-O, Cipriani AA-O: **Combining endpoint and change data did not affect the summary standardised mean difference in pairwise and network meta-analyses: An empirical study in depression. LID - 10.1002/jrsm.1719 [doi]**. 2024(1759-2887 (Electronic)).
19. Furukawa TA, Barbui C Fau - Cipriani A, Cipriani A Fau - Brambilla P, Brambilla P Fau - Watanabe N, Watanabe N: **Imputing missing standard deviations in meta-analyses can provide accurate results**. 2006(0895-4356 (Print)).
20. deSouza IS, Thode HC, Jr., Shrestha P, Allen R, Koos J, Singer AJ: **Rapid tranquilization of the agitated patient in the emergency department: A systematic review and network meta-analysis**. *Am J Emerg Med* 2022, **51**:363-373.
21. Baldaçara L, Pinto ALdCB, Díaz AP, Sanches M, da Silva AG: **Rapid tranquilization in a psychiatric emergency room: A naturalistic cohort study in 12 h**. *Psychiatry Research Communications* 2024, **4**(2):100168.
22. Baldaçara L, Diaz AP, Leite V, Pereira LA, Dos Santos RM, Gomes VdP, Calfat EL, Ismael F, Périco CA, Porto DM: **Brazilian guidelines for the management of psychomotor agitation. Part 2. Pharmacological approach**. *Brazilian Journal of Psychiatry* 2019, **41**:324-335.
23. Roppolo LP, Morris DW, Khan F, Downs R, Metzger J, Carder T, Wong AH, Wilson MP: **Improving the management of acutely agitated patients in the emergency department through implementation of Project BETA (Best Practices in the Evaluation and Treatment of Agitation)**. *J Am Coll Emerg Physicians Open* 2020, **1**(5):898-907.

Treatment of severe agitation in real-world settings: a protocol of individual-participant-data network meta-analysis

To enable PROSPERO to focus on COVID-19 submissions, this registration record has undergone basic automated checks for eligibility and is published exactly as submitted. PROSPERO has never provided peer review, and usual checking by the PROSPERO team does not endorse content. Therefore, automatically published records should be treated as any other PROSPERO registration. Further detail is provided here.

Citation

Spyridon Siafis, Johannes Schneider-Thoma, Irene Bighelli, Clive E. Adams, Joseph E Dib, Prathap Tharyan, Leonie A Calver, Geoffrey K Isbister, Esther WY Chan, Jonathan C Knott, Celene YL Yap, Célia Mantovani, Marc L Martel, Wulf-Peter Hansen, Georgia Salanti, Stefan Leucht. Treatment of severe agitation in real-world settings: a protocol of individual-participant-data network meta-analysis. PROSPERO 2023 CRD42023402365 Available from: https://www.crd.york.ac.uk/prospero/display_record.php?ID=CRD42023402365

Review question

To investigate the comparative efficacy and tolerability of intramuscular and intravenous pharmacological interventions for severe agitation in real-world settings.

Searches

1. Electronic databases, i.e., MEDLINE (via Ovid), Embase (via Ovid), PsycINFO (via Ovid), PubMed, CENTRAL, CINAHL, LILACS, BIOSIS, ProQuest.

2. Clinical trial registries, i.e., ClinicalTrials.gov and WHO-ICTRP.

3. Inspection of the reference lists of previous reviews, e.g., (Ostinelli et al., 2017; Zaman et al., 2017) and of all included studies.

4. The first author of each included study and pharmaceutical companies of industry-sponsored studies will be conducted for additional relevant studies and for requesting de-identified individual-participant-data (IPD).

There will be no restriction in terms of language and publication status. However, we will search for studies that were published since 2002 (last 20 years) (see “Types of study to be included”).

Search strategy

https://www.crd.york.ac.uk/PROSPEROFILES/402365_STRATEGY_20230223.pdf

Types of study to be included

Randomized-controlled trials (RCTs) comparing at least 2 eligible interventions (see "Interventions/exposures") in patients with severe psychomotor agitation and/or aggression, and assessing sedation up to a maximum of 30 minutes (see “Main outcome”).

Eligible RCTs would be conducted in emergency settings (see "Context").

We will include both open and blinded (single-, double-blind) RCTs, but studies with a high risk of bias in the randomization process will be excluded (see “Risk of bias”). In case of crossover trials, we will use only the first phase to avoid carry-over effects. In case of cluster-randomised trials, we will include them but we will adjust for "unit-of-analysis" issues.

Moreover, we will include studies that were published since 2002 due to the difficulties in the retrieval of IPD from older trials, and the potential differences in their design and quality compared with more recent trials.

Condition or domain being studied

Severe psychomotor agitation and aggression.

Participants/population

Adults participants with severe agitation and/or aggression requiring emergency treatment with parenteral medications, who should be unable to cooperate and provide informed consent (see “Types of studies included”). Agitation is a transdiagnostic condition and emergency treatment often precedes formal diagnosis, and thus, there will be no restriction in terms of underlying diagnosis and diagnostic criteria. However, studies focusing on a specific underlying diagnosis of agitation such as schizophrenia or dementia will be excluded (see “Types of studies included”). We will also exclude children and adolescents, and there will be no other restriction in terms of age, sex, and ethnicity.

Intervention(s), exposure(s)

We will include intramuscular (i.m.) and intravenous (i.v.) medications that are used in monotherapy and/or combination to calm patients with agitation. We will consider i.m. and i.v. formulations as separate nodes. A long list of medications will be eligible such as antipsychotics (e.g., haloperidol, olanzapine, promethazine), antihistamines (e.g., diphenhydramine), benzodiazepines (e.g., lorazepam, midazolam), alpha2 adrenergic agonist (e.g., clonidine, dexmedetomidine). There will be no restriction in terms of dose, which will be investigated as covariate in the model (see “Strategy for data synthesis”). However, we will exclude barbiturates, i.e., old medications with narrow therapeutic window that are no longer used for agitation. We will also exclude oral and inhaled administration because they require co-operation of the participants, and this would have violated the transitivity assumption (see “Participants”).

Comparator(s)/control

In a network meta-analysis, each intervention will be compared with each other. We will exclude placebo as a comparator (see “Type of studies included”), and haloperidol will be used as reference in forest plots.

Context

Eligible RCTs would be conducted in emergency settings, i.e., general or psychiatric emergency rooms, and the requirement of the participants to provide informed consent before the study start would have been waived. Thus, we will exclude registration trials and placebo-controlled RCTs that require informed consent of the participants or the legal guardians before the study start. When RCTs required informed consent from the legal guardians or the participants after the study start, they will be included only if <10% of the participants are lost because of this in order to avoid selection bias.

Main outcome(s)

The primary outcome will be the number of participants with adequate sedation. There are various ways and scales for measuring sedation and/or agitated behavior (e.g., Sedation Assessment Tool, SAT) that could be used across studies. We will use IPD to apply relatively homogeneous cutoffs and definitions of adequate sedation, i.e., calm or asleep but not over-sedated.

Measures of effect

The effect size will be odds ratios (ORs) presented with their 95%CIs. The primary timepoint will be at latest at 30 minutes (preferably at 20 minutes) after the first administration of the pharmacological intervention. We will also evaluate secondary timepoints at 10 minutes, 30 minutes, 45 minutes, 2 hours, 4 hours and 24 hours after the first administration of the pharmacological intervention.

Additional outcome(s)

1. Time to adequate sedation.

2. Number of patients requiring additional medication.

3. Number of patients requiring physical restraint.

4. Mean scores of rating scales measuring agitated behavior, e.g., Positive and Negative Syndrome Scale Excitement Component (PANSS-EC), Overt Aggression Scale (OAS).

5. Time to discharge from the hospital.

6. Number of patients with important side-effects of the eligible medications, i.e., seizures, dystonia, akathisia, parkinsonism, any extrapyramidal side-effect, need for antiparkinsonian medications, QTc interval prolongation and arrhythmias, falls, respiratory depression, aspiration, allergic reaction, bronchospasm, oversedation, hypotension, nausea and vomiting. Side-effects could be reported in various ways across trials, and we will harmonize them by using MedDRA ontology.

7. Mean scale scores of extrapyramidal side-effects, e.g., Simpson Agnus Scale (SAS), Barnes Akathisia Rating Scale (BARS).

8. Number of patients that died for any reason.

9. Number of patients with premature discontinuation due to any reason, inefficacy and adverse events.

Measures of effect

The effect size for continuous outcomes will be the standardized mean difference (SMD) and for dichotomous outcomes will be the ORs, presented with their 95%CI.

Data extraction (selection and coding)

1. Study selection: At least two reviewers will independently inspect the titles/abstracts in a first step, and full-texts of relevant records in a second step. Disagreements will be resolved by consulting a third senior reviewer of by requesting further information by study authors.

2. IPD dataset: The principal investigators of eligible trials will be requested to join the project and share de-identified IPD of their studies. We will request for data about study design, participant and intervention characteristics as well as outcome measures for all available timepoints. We will homogenize the variables across individual trials in a common format and construct a single dataset. We evaluate the integrity of IPD by checking for missing, outlier and duplicated values, if randomization was appropriate and by cross-checking with the summary statistics of the published reports. Any issue will be resolved with the study authors.

3. Data extraction of aggregated data: When IPD are not available for eligible trials, two independent reviewers will extract aggregated data in a Microsoft Access database. Disagreements will be resolved by consulting a third senior reviewer of by requesting further information by study authors. We will follow standard approaches as in our previous reviews, i.e., giving preference to extracting data from all randomized participants using appropriate imputation or modelling methods over last-observation-carried forward or extracting data from completers or compliers only, giving preference to change over endpoint scores as well as deriving missing standard deviation according to the Cochrane Handbook.

Risk of bias (quality) assessment

Two independent reviewers will evaluate the risk of bias of eligible trials using the Cochrane risk of bias tool version 2, which considers the domains of randomization process, deviations from intended interventions, missing outcome data, measurement of the outcome, and selection of reported results. We will use the information provided by the IPD to assess the risk of bias of the studies with IPD .

Strategy for data synthesis

We will synthesize the data using multilevel network meta-regression models in a Bayesian framework (Donegan et al., 2013; Hamza et al., 2023; Saramago et al., 2012) using the R package crossnma (Hamza et al., 2022; Hamza et al., 2023). We will opt to obtain IPD for all eligible studies, yet we will aim to combine IPD and aggregated data, if the former are not available. We will consider including potential prognostic factors and effect-modifiers as covariates in the model (see “Analysis of subgroups”). Missing outcome and covariate data will be imputed with multiple imputations by assuming missing at random (MAR), whenever appropriate, and accounting the stratification of patients in trials. We will use minimally informative prior for effect-sizes and regression coefficients, and half-normal for between-study variance (τ2).

Heterogeneity will be quantified using the τ2, which will be assumed to be common across treatment comparisons.

The transitivity assumption of the network meta-analysis will be examined by comparing the distribution of potential effect-modifiers across treatment comparisons. The statistical manifestation of intransitivity (incoherence), will be examined with local, i.e., SIDE test, and global approaches, i.e., design-by-treatment interaction test.

Small-study effects will be evaluated with a meta-regression using the study variance as covariate.

We will evaluate the confidence in the evidence using the CINeMA framework (Nikolakopoulou et al., 2020).

Analysis of subgroups or subsets

1. We will examine the following predefined covariates as potential prognostic factors and effect-modifiers: age, sex, ethnicity, baseline severity of agitation, diagnostic subgroups, medication use before injection, setting, and dose.

2. We will conduct the following predefined sensitivity analysis to investigate the robustness of the findings for the primary outcome: 1) exclusion of open and single-blinded studies and 2) exclusion of studies with an overall high risk of bias.

3. Data availability bias will be explored by examining potential differences between studies with available and unavailable IPD in terms of their study design and participant characteristics, and their effect sizes.

Contact details for further information

Dr. Spyridon Siafis

spyridon.siafis@tum.de

Organisational affiliation of the review

Department of Psychiatry and Psychotherapy, School of Medicine, Technical University of Munich, Munich, Germany

https://ebmpp.org/de

Review team members and their organisational affiliations

Dr Spyridon Siafis. Department of Psychiatry and Psychotherapy, School of Medicine, Technical University of Munich, Munich, Germany

Dr Johannes Schneider-Thoma. Department of Psychiatry and Psychotherapy, School of Medicine, Technical University of Munich, Munich, Germany

Dr Irene Bighelli. Department of Psychiatry and Psychotherapy, School of Medicine, Technical University of Munich, Munich, Germany

Professor Clive E. Adams. Institute of Mental Health, University of Nottingham, Nottingham, Nottinghamshire, UK

Dr Joseph E Dib. Division of Psychiatry & Applied Psychology, Institute of Mental Health, School of Medicine, University of Nottingham, Nottingham, Nottinghamshire, NG1 1NU, UK

Professor Prathap Tharyan. Clinical Epidemiology Unit, Christian Medical Centre, Vellore, India.

Dr Leonie A Calver. School of Nursing and Midwifery, University of Newcastle, Callaghan, New South Wales, Australia.

Professor Geoffrey K Isbister. Clinical Toxicology Research Group, University of Newcastle, Newcastle, New South Wales, Australia

Professor Esther WY Chan. Department of Pharmacology and Pharmacy, University of Hong Kong, Hong Kong

Professor Jonathan C Knott. Department of Critical Care, The University of Melbourne, Melbourne, Victoria, Australia

Dr Celene YL Yap. Department of Nursing, Melbourne School of Health Sciences, Faculty of Medicine, Dentistry and Health Sciences, The University of Melbourne, Melbourne, Victoria, Australia

Professor Célia Mantovani. University of New England, Portland, Oregon, USA

Professor Marc L Martel. Department of Emergency Medicine, Hennepin County Medical Center, Minneapolis, Minnesota, USA

Mr Wulf-Peter Hansen. BASTA - Bündnis für psychisch erkrankte Menschen, Munich, Germany

Professor Georgia Salanti. Institute of Social and Preventive Medicine, University of Bern, Bern, Switzerland.

Professor Stefan Leucht. Department of Psychiatry and Psychotherapy, School of Medicine, Technical University of Munich, Munich, Germany

Collaborators [1 change]

Dr Farhad Shokraneh. Systematic Review Consultants LTD

Type and method of review

Individual patient data (IPD) meta-analysis, Intervention, Network meta-analysis, Systematic review

Anticipated or actual start date

01 December 2022

Anticipated completion date

01 February 2025

Funding sources/sponsors

Federal Ministry of Education and Research (Bundesministerium für Bildung und Forschung)

Grant number(s)

State the funder, grant or award number and the date of award

01KG2208

Conflicts of interest

In the last three years Stefan Leucht has received honoraria as a consultant and/or advisor and/or for lectures and/or for educational material from Alkermes, Angelini, Eisai, Gedeon Richter, Janssen, Lundbeck, Medichem, Medscape, Merck Sharpp and Dome, Mitshubishi, Neurotorium, NovoNordisk, Otsuka, Recordati, Roche, Rovi, Sanofi Aventis, TEVA. No conflicts of interest were declared by the other authors.

Yes

Language

English

Country

Germany

Stage of review

Review Ongoing

Subject index terms status

Subject indexing assigned by CRD

Subject index terms

Anxiety; Humans; Meta-Analysis as Topic; Network Meta-Analysis; Psychomotor Agitation

Date of registration in PROSPERO

07 March 2023

Date of first submission

24 February 2023

Stage of review at time of this submission

Stage	Started	Completed
Preliminary searches	Yes	Yes
Piloting of the study selection process	No	No
Formal screening of search results against eligibility criteria	No	No
Data extraction	No	No
Risk of bias (quality) assessment	No	No
Data analysis	No	No

Revision note

The affiliation of Dr. Farhad Shokraneh was corrected to Systematic Review Consultants LTD.

The record owner confirms that the information they have supplied for this submission is accurate and complete and they understand that deliberate provision of inaccurate information or omission of data may be construed as scientific misconduct.

The record owner confirms that they will update the status of the review when it is completed and will add publication details in due course.

Versions

07 March 2023
07 March 2023
09 March 2023
